# The influence of the negative-positive ratio and screening database size on the performance of machine learning-based virtual screening

**DOI:** 10.1371/journal.pone.0175410

**Published:** 2017-04-06

**Authors:** Rafał Kurczab, Andrzej J. Bojarski

**Affiliations:** Department of Medicinal Chemistry, Institute of Pharmacology, Polish Academy of Sciences, Kraków, Poland; Shool of Pharmaceutical Sciences, Sun Yet-Sen University, 132 Easy Cycle at University City, CHINA

## Abstract

The machine learning-based virtual screening of molecular databases is a commonly used approach to identify hits. However, many aspects associated with training predictive models can influence the final performance and, consequently, the number of hits found. Thus, we performed a systematic study of the simultaneous influence of the proportion of negatives to positives in the testing set, the size of screening databases and the type of molecular representations on the effectiveness of classification. The results obtained for eight protein targets, five machine learning algorithms (SMO, Naïve Bayes, Ibk, J48 and Random Forest), two types of molecular fingerprints (MACCS and CDK FP) and eight screening databases with different numbers of molecules confirmed our previous findings that increases in the ratio of negative to positive training instances greatly influenced most of the investigated parameters of the ML methods in simulated virtual screening experiments. However, the performance of screening was shown to also be highly dependent on the molecular library dimension. Generally, with the increasing size of the screened database, the optimal training ratio also increased, and this ratio can be rationalized using the proposed cost-effectiveness threshold approach. To increase the performance of machine learning-based virtual screening, the training set should be constructed in a way that considers the size of the screening database.

## Introduction

Machine learning (ML) methods are widely used in drug discovery to classify molecules as potentially active or inactive against a particular protein target. The vast majority of those methods require the preparation of a training set of compounds (supervised learning) that are used to develop a decision function that can be used for virtual screening (VS) of chemical libraries among particular activity classes [1]. The role of machine learning in drug design has been the subject of numerous studies regarding optimal learning parameters and examining their impact on classification effectiveness [2–5], comparing the performance of different ML algorithms in virtual screening [4,5] and learning from imbalanced data [6–8].

In fact, the number of compounds in a screening library that is used in virtual screening is strictly determined by the source of the compounds. Thus, the size of the screening library can vary from several hundred, especially in the case of in-house, reaction-based combinatorial libraries, to millions of compounds, which are available from commercial suppliers. Recently, several analyses and evaluations of compound libraries from commercial suppliers have been published [9–13]. Following the results of Petrova et al., vendors can be divided into three groups according to the size of the libraries that they provide: less than 100,000 compounds (15 suppliers), from 100,000 to 500,000 compounds (11 suppliers) and more than 500,000 compounds (10 suppliers) [13]. The authors also noted that the highest percentage of exclusive compounds was found for the first (90%) and the second group (~50%). Based on these outcomes and taking into account practical aspects of virtual screening, we focused our study on databases from the first two classes.

It was recently shown that ML classification effectiveness depended on the inactive set design and the ratio of negative to positive training examples [14,15]. Here, the relationship between the size of a screening database and the effectiveness of ML-based virtual screening was systematically studied. First, the influence of the proportion of negative to positive examples in the training set on screening performance was assessed for different testing set sizes, and second, an approach to rationalize the choice of the training ratio was proposed.

## Materials and methods

### Compound data sets

The ChEMBL (version 18) Target Classification Hierarchy directed the selection of the eight targets used in the tests, which ensured the diversity of both the proteins and structures of the active compounds: 5-HT_1A_R agonists, HIV-1 protease inhibitors (HIV Pr), SERT inhibitors, estrogen receptor alpha agonists (ER-α), acetylcholinesterase inhibitors (AChE), phosphodiesterase 5A inhibitors (PDE5), cyclin-dependent kinase 2 inhibitors (CDK2) and corticotropin-releasing factor receptor 1 (CRFR1). As ChEMBL contains numerical values of particular parameters that determine the activity of the compounds, only molecules whose activities were quantified by *K*_i_, p*K*_i_ or IC_50_ and were tested in human protein assays were taken into account. The p*K*_i_ and IC_50_ values were recalculated to *K*_i_ using the following expressions: *K*_i_ = 10^–p*K*i^ and *K*_i_ = IC_50_/2 (the conversion factor of 2 was suggested by Kalliokoski et al. [16]). The compounds were considered to be active when the *K*_i_ value was lower than 100 nM.

ML models were built and tested using active compounds and assumed inactive compounds that were randomly selected from ZINC v. 11 (details presented in Table 1) [17]. Because different numbers of active ligands were obtained, the chosen number of inactives was rescaled to ensure the same active to inactive ratios varying from 0.5 to 100. The positive training set was fixed and composed of approximately 18% of all of the compounds that had confirmed activity toward a particular target. The test sets (screening databases) with different sizes (i.e., 5 k, 10 k, 25 k, 50 k, 75 k, 100 k, 200 k and 400 k) were formed by merging the remaining actives together with the appropriate number of compounds randomly selected from ZINC. For each ratio of inactive to active compounds and screening database size, 10 trials were performed.

**Table 1:** Composition of the training and test sets used.

Target	ChEMBL class	ChEMBL target ID	Number of actives	
			Training set	Test set
**5-HT**_**1A**_**R**	membrane receptor	CHEMBL214	198	903
**HIV Pr**	enzyme/protease	ChEMBL243	203	932
**SERT**	transporter	CHEMBL228	390	1822
**ER-α**	nuclear receptor	CHEMBL206	133	614
**AChE**	enzyme/hydrolase	CHEMBL220	162	743
**PDE5**	enzyme/phosphodiesterase	CHEMBL1827	152	695
**CDK2**	enzyme/kinase	CHEMBL301	236	1084
**CRF1**	membrane receptor	CHEMBL1800	200	914

The changes in recall, precision and MCC values between particular iterations were statistically insignificant, and therefore, repeating the study with another randomly selected ZINC set led to very similar results, and the dependencies connected with the number of inactives in the training set were preserved.

### Machine learning algorithms

Five of the most commonly used cheminformatics ML algorithms were selected: Sequential Minimal Optimization (SMO) [18], Naïve Bayes classifier (NB) [19], Instance-Based Learning (Ibk) [20,21], J48 [22] and Random Forest (RF) [23,24]. All machine learning calculations were carried out using the WEKA package (version 3.6)[25]. The default settings of all of the tested classifiers were applied (see Table 2).

**Table 2:** Machine learning algorithms used and a short description of their training parameters.

Classifier	Classification scheme	Settings
**Sequential Minimal Optimization (SMO)**^a^	functions	The complexity parameter was set at 1, the epsilon for a round-off error was 1.0 E-12, and the option of normalizing training data was chosen. The normalized polynomial kernel was used.
**Naïve Bayes (NB)**	bayes	–
**Instance-Based Learning (Ibk)**^b^	lazy	The nearest neighbor search algorithm using the Euclidean distance function and 1 neighbor.
**J48**^c^	trees	C.4.5 pruning
**Random Forest (RF)**	trees	Trees with unlimited depth, seed number: 1. Number of generated trees: 10.

^a^the SVM algorithm implemented in WEKA,

^b^the *k*-NN algorithm implemented in WEKA,

^c^the decision tree algorithm implemented in WEKA.

### Molecular descriptors

The subsets of compounds fetched from ChEMBL were standardized using the ChemAxon Standardizer [26] with the following options: Remove Fragment, Neutralize, RemoveExplicitH, Clean2D, Mesomerize and Tautomerize. The standardized sets were next cleaned for compounds that were too small or too large (200 Da < MW < 700 Da) and checked for duplicate ligand structures. The obtained compound structures were represented by using MACCS structural keys [27] and CDK standard hashed fingerprints with a default path length of 6 (FP) [28]; they were generated by PaDEL-Descriptor software [29].

### Calculations and performance measures

The evaluation of the ML-based virtual screening performance was executed with the following parameters (averaged over 10 trials): recall–R Eq (1), precision–P Eq (2) and Mathews Correlation Coefficient–MCC Eq (3):

$$R=\frac{TP}{TP+FN}$$ (1)

$$P=\frac{TP}{TP+FP}$$ (2)

$$MCC=\frac{TP\cdot TN-FP\cdot FN}{\sqrt{\left(TP+FP)\cdot (TP+FN)\cdot (TN+FP)\cdot (TN+FN\right)}}$$ (3)

Recall measures the number of correctly identified positive instances, precision describes the correctness of positive predictions and MCC is a balanced measure of binary classification effectiveness, ranging from –1 to 1, with 1 referring to perfect prediction.

These parameters were selected to enable the assessment of the classification effectiveness from various perspectives. All experiments were performed on an Intel Core i7 CPU 3.00 GHz computer system with 24 GB RAM running a 64-bit Linux operating system using in-house scripts.

## Results and discussion

The main objective of this study was to determine how the optimal ratio of inactive to active (IN/A) training instances depends on the screening database size in machine learning-based screening of molecular databases. To address this issue, calculations were performed for eight protein targets (Table 1) belonging to different classes (enzymes, membrane proteins, transcription factors, transporters) and for compounds stored in the ChEMBL database [30]. Two types of molecular fingerprints (MACCS and CDK FP) were applied to build the training datasets of a fixed number of positive instances and the number of negative examples was varied (to obtain 17 IN/A training ratios, ranging from 0.5 to 100). Five machine learning algorithms (Sequential Minimal Optimization–SMO, Naïve Bayes–NB, Ibk, J48 and Random Forest–RF) were used in the screening of eight screening libraries whose magnitudes were established to reflect the commercial collections of available compounds and combinatorial libraries that are often used in virtual screening [13].

The performance of ML-based screening was assessed with the use of recall, precision, Matthews Correlation Coefficient (MCC) and Precision-Recall (PR) plots, which are usually used to provide comprehensive assessments of imbalanced learning problems [8,31]. Additionally, the two-way ANOVA was conducted (for details see S2 File) to evaluate the different effects (target, ML algorithm, fingerprint type and screening library size) on the global performance of virtual screening (MCC).

### Influence of the negative training set size on the performance of ML methods

**Fig 1:**
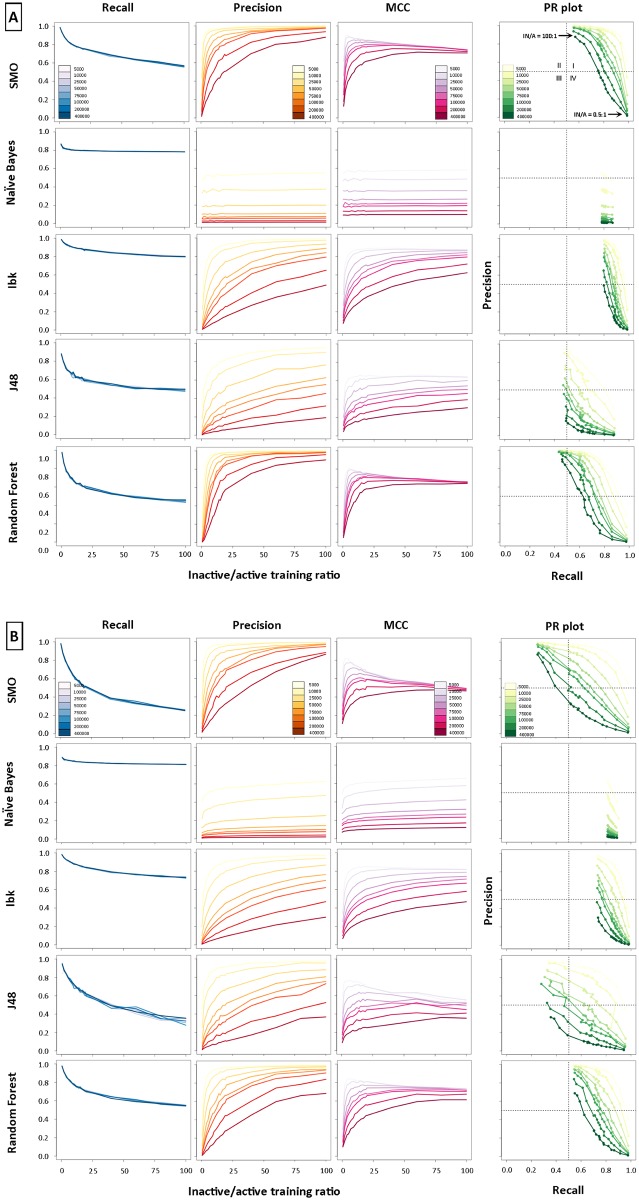
The dependence of the negative training set size on machine learning-based virtual screening performance for 2 types of fingerprints (panel A–CDK FP, and MACCS FP in B) averaged over 10 independent trials. The colored lines denote the type of evaluated parameter used (blue–recall, red–precision, magenta–MCC and green–PR plot).

The results obtained for 5-HT_1A_R are presented in Fig 1 (panel A for CDK FP and B for MACCS FP), showing recall, precision, MCC and PR plots for five ML methods and eight screening libraries of different sizes (5000–400,000 compounds); data for the remaining protein targets are available in the Supporting Information (S1 Fig). A single plot illustrates the relation between the average (after 10 iterations) value of a given performance measure, which was calculated for a combination of the IN/A training ratio and the set of screening databases used.

Global analysis confirmed our previous findings [15] that increasing the ratio of IN/A training examples significantly improved the effectiveness of ML-based virtual screening as well as the different behavior of the Naïve Bayes algorithm, which in all cases showed only slight sensitivity to the enlargement of the negative training set size (Fig 1). According to NB methodological assumptions, instances from the test set are labeled according to the class distribution from the training data. Therefore, one would expect that increasing the number of inactive compounds in the training set would lead to improvement of the Naïve Bayes performance in a virtual screening-like experiment. However, attempts to reproduce the class distribution from the training set led to errors in class assignments for sets with a higher number of inactives, which in turn resulted in lower values of the evaluating parameters instead of the expected increase in values. The remaining ML methods (SMO, Ibk, J48 and RF) aimed at maximizing the overall accuracy of the objective function (the ratio of the number of true predictions–the sum of TP and TN, out of all predictions made). Hence, when the IN/A training ratio increases, the majority classifier is produced, which leads to an over-prediction of the presence of the majority (negative) class.

Recall decreases when the IN/A training ratio increases, except for NB for which after small decrease a constant level was observed. Moreover, increasing the dimension of the screening database did not influence recall. For a particular IN/A training ratio, recall showed almost the same value in all of the screened libraries. These observations can be explained by means of the expression for recall (eq 1). The positive instances in training and testing sets (true positives, TP), as well as negative sets included in the screening library (true negatives, TN), were fixed, and only the negative training examples were changed. Because recall calculates only the classification of positive instances (active compounds can be classified as TP or FN) and does not count assumed inactive classifications (FP or TN), its value will not change, even when the screening databases increase in size. In larger databases, only more false positives (FP) and true negatives (TN) can be found, which are not used in recall calculations. Furthermore, adding more negative training examples leads to classifiers that over-predict the negative class from the screening database, which can consequently produce an incorrect classification of true positives (as FN) and thereby a decrease in recall.

Precision improves (Fig 1, S1 Fig) when the IN/A training ratio increases and simultaneously deteriorates with an enlargement of the screening library. However, for some combinations of protein target and the ML method, precision showed interesting features. It reached a maximum value very quickly (for low IN/A training ratio) that did not change even when the IN/A training ratio increased, and the reduction in precision from the enlargement of the screening database seemed to be less significant (e.g., HIV Pr, Er-α). Again, the obtained trends can be explained with respect to the expression of precision–it only counts the number of correctly classified actives (TP) and incorrectly classified inactives (FP) from the screening library. Increasing the number of inactive compounds in the training set causes better recognition of negative examples by the ML objective function and thus an increase in incorrectly classified actives (this remark is in line with the results obtained for recall) and improved classification of inactives (counted as TN).

In addition, analysis of the precision-recall plots showed that initially, all of the models had a medium classification effectiveness with high recall and low precision (panel A, quarter IV in PR plot, Fig 1). When the size of the negative set increased (panel A, the PR plot for SMO, Fig 1), performance improvements were observed for all methods except Naïve Bayes (S1 Fig). The most significant changes were found for the SMO, Ibk and RF methods, which moved to the region of high recall and precision (quarter I). Considering the dynamics of the changes in ML performance with a growing number of negative training examples, the SMO and RF algorithms quickly led to models that had very good classification effectiveness (panel A, Fig 1). In comparison, the improvement of the J48 method was less significant, and the corresponding curves on the precision-recall plots responded very slowly to the increase in the number of negative instances. Interestingly, increasing the size of the screening library caused a slower shift to the area corresponding to good models (quarter I), and in some cases (MACCS in combination with SMO or J48 and database size = 100 k and 200 k, respectively), this region was even omitted and medium models were obtained with low recall and high precision (panel B, quarter III, Fig 1).

### Rational choice of the IN/A training ratio for ML-based virtual screening

**Fig 2:**
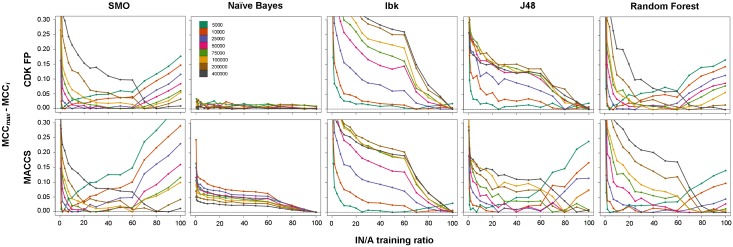
The dependency of the IN/A training ratio on the cost-effectiveness thresholds for different screening library sizes.

In our previous study [15], we concluded that the preferable ratio of inactive to active compounds in the training sets was approximately 9:1–10:1, and only slight improvements in global ML methods performance were observed by further increasing of the negative training set size. Moreover, we noted that the indicated preferable IN/A training ratio might change under different experimental conditions, such as the dimension of the screening database. We now explored this issue by performing experiments on screening libraries of diverse sizes (5 k, 10 k, 25 k, 50 k, 75 k, 100 k, 200 k and 400 k) and with different IN/A training ratios (ranging from 0.5 to 100). Additionally, we observed that increasing the number of negative training examples was not profitable due to increases in computational expenses, which was even more prominent for larger libraries. Thus, we propose the strategy of searching for the optimal IN/A training ratio with respect to the type of machine learning algorithm and size of a screening library used. Fig 2 shows the dependency of the IN/A training ratio on cost-effectiveness, expressed as the difference between the best MCC found in the screening of a particular database and the MCC calculated for each training ratio. Initially, increasing the negative training examples led to improved MCC values up to the IN/A training ratio corresponding to the best MCC, whereas a further increase in negative examples (except J48 and Ibk) caused a decline in model performance. No significant changes were recorded for NB, which is in line with previously described observations.

**Fig 3:**
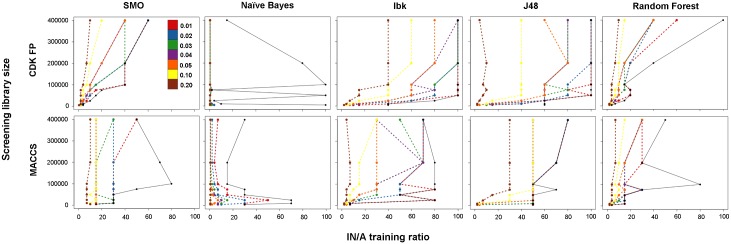
The dependency of the optimal IN/A training ratio from the size of the screening library obtained for several arbitrarily selected cost-effectiveness thresholds. For comparison, the training ratio obtained for the best MCC was added (black line).

It should be noted that when approaching the optimal IN/A training ratio (i.e., that corresponded to the highest MCC value), there are some ratios with only slightly lower MCC values. Thus, for several cost-effectiveness thresholds (calculated as the distance to the best MCC value), the IN/A training ratios were minimized for different sizes of screening databases (Fig 3). To show the level of reduction, the training ratio obtained for the best MCC was added (black line in Fig 3).

The results clearly show that the proposed approach can be effectively used to reduce the negative to positive training set size. This reduction is strictly related to the cost-effectiveness threshold used. In general, when the cost-effectiveness threshold increased, the reduction of the IN/A training ratio increased as well. However, when the screening database increased, the observed reduction was more significant, with larger distances between black and corresponding dashed lines (Fig 3). Here, it should be noted, that Naïve Bayes was found to be the most sensitive to the reduction approach that was used and showed the largest training ratio decrease with an increasing threshold.

The minimized training ratios for all eight protein targets were obtained for the smallest (5 k), medium (50 k) and the largest (400 k) screening databases and for all combinations of molecular fingerprints and ML methods using a cost-effectiveness threshold equal of 0.03, as summarized in Table 3 (the full results are available in S1 Table).

**Table 3:** The optimal IN/A training ratios obtained for a cost-effectiveness threshold equal 0.03.

Target	Screening library size	Best IN/A ratio									
		SMO		NB		Ibk		J48		RF	
		CDK FP	MACCS	CDK FP	MACCS	CDK FP	MACCS	CDK FP	MACCS	CDK FP	MACCS
**5-HT**_**1A**_**R**	5000	2	2	2	60	10	10	10	2	4	4
	50000	7	7	0.5	40	60	80	60	15	10	15
	400000	40	40	0.5	10	100	100	80	80	40	60
**HIV Pr**	5000	2	2	0.5	4	4	4	7	4	4	4
	50000	4	10	4	10	10	15	40	40	7	15
	400000	10	40	2	2	40	40	80	80	20	60
**SERT**	5000	1	1	0.5	0.5	4	1	2	1	1	1
	50000	2	4	0.5	10	20	10	10	4	2	7
	400000	7	20	0.5	7	30	30	30	20	7	20
**ER-α**	5000	4	4	1	15	7	7	7	7	4	4
	50000	7	15	7	25	30	60	60	90	15	25
	400000	7	90	7	7	60	90	60	90	15	90
**AChE**	5000	2	2	2	50	10	4	10	10	4	5
	50000	7	10	2	50	50	15	70	70	10	25
	400000	10	50	4	2	70	100	100	70	15	100
**PDE5**	5000	2	2	0.5	4	4	10	15	10	4	4
	50000	7	10	0.5	50	20	50	100	50	10	20
	400000	10	50	2	10	50	100	80	100	15	100
**CDK2**	5000	2	15	2	7	4	4	7	4	4	4
	50000	4	15	2	4	30	30	80	50	7	15
	400000	7	30	0.5	0.5	50	50	50	50	10	30
**CRF1**	5000	2	2	2	10	4	4	4	4	4	4
	50000	7	10	2	40	40	60	60	40	10	20
	400000	40	80	0.5	7	60	80	80	80	40	80

The results are consistent for all of the studied proteins and show that increasing the size of the screening database causes an increase in the optimal (with the assumption that a difference of the best MCC of 0.03 is acceptable) negative to positive training ratio. However, for the ML algorithms used, the level of training set increase was diverse. The lowest increase was detected for SMO and RF (approximately 2–40 for CDK FP), whereas the highest increase was found for Ibk and J48 (approximately 2–100 for MACCS FP). Completely different performances were observed for a combination of CDK FP and NB, for which no significant changes in the optimal training ratio from increasing the screening database size were observed (in the majority of cases, the optimal IN/A training ratio was 0.5:1), and no clear trend was found for MACCS FP (disordered). Interestingly, the combination of Naïve Bayes and CDK FP showed, globally, (S1 Table, S3 Fig) the lowest optimal IN/A training ratios, but simultaneously, the worst overall performance (MCC).

### Target dependency

In general, these conclusions were consistent for all of the protein targets, but a slight influence of the target type on the performance of virtual screening was observed. Additional calculations showed that the ligand chemotype diversity of a given target and the density of the screening compounds that had a high similarity to the active compounds may be essential in explaining target dependency (for details, see S1 File). Moreover, the results of the two-way ANOVA indicated (see S2 File: Case 1, 2 and 5) a significant main effect for FP type (p < 0.0001), ML method (p < 0.0001) and screening database size (p < 0.0001) on the performance of virtual screening (given by MCC). Additionally, the results showed a significant interaction between protein targets and mentioned effects (p < 0.0001).

### Fingerprint dependency

In almost all cases, the total improvement in the predictive models was clearly better for CDK FP than MACCS FP (Fig 1 and S1 Fig). Additionally, this was confirmed by precision-recall plots, where the performance of a particular ML algorithm changed more dynamically when molecules were encoded by CDK FPs than MACCS fingerprints in almost all cases studied. The results of the two-way ANOVA confirmed (see S2 File: Case 3 and 4) that there was a significant difference among the virtual screening performance for CDK FP and MACCS (p < 0.0001). Interestingly, the interaction plots showed that the difference between mean values of MCC for CDK FP and MACCS had no effect for 5-HT_1A_R and SERT targets (S2 File, Case 2) and J48 ML method (S2 File, Case 3).

By searching for the optimal IN/A training ratio using different cost-effectiveness thresholds, in almost all cases studied, a lower training ratio was found for CDK FP (S1 Table).

## Conclusions

In this study, we investigated the performance of a collection of machine learning algorithms in ligand-based virtual screening in cases in which the inactive to active training ratio and screening library size were iteratively changed. We found that increasing the size of the negative training set (with a constant number of positives) led to a decrease in recall and an improvement in precision and MCC. The results were consistent for all protein targets and fingerprints and were in line with results from previous reports [15,32]. However, it should be noted that the optimal IN/A training ratio, speed of achieving the maximal performance (precision, MCC), and decrease in performance (precision, MCC) with an increasing screening database size are target-dependent. We suggested that similarity (pairwise similarity) between active and screening compounds may be essential in explaining target dependency.

According to the use of different sizes of compound databases in ML-based virtual screening, we found that the searching performance was very diverse. Generally, increasing the number of compounds in the screening library deteriorated the precision and MCC and did not change the recall. The second outcome revealed that, except for Naïve Bayes, the IN/A training ratio for which the best MCC was observed increased with the increasing the size of the screening library. All these outcomes were validated by the two-way ANOVA which showed a significant interaction between screening library size with protein target (S2 File, Case 5) and fingerprint type (S2 File, Case 4).

Enlargement of the training ratio leads to an increase of the time needed for training prediction models and, consequently, for searching molecular libraries. Thus, we proposed a rationalization strategy of selecting the optimal training set size. Using self-defined cost-effectiveness thresholds (difference between the best MCC and remaining MCCs obtained for screening of a particular database), we showed that a many-fold lower IN/A training ratio can be used to build a predictive model with only a marginal drop in MCC value compared to the best value obtained when no training ratio constraint was used. The lowest training ratio (cost-effectiveness threshold equal 0.03) was obtained for a combination of Naïve Bayes and CDK FP (0.5:1–4:1), but simultaneously, the overall performance was the worst (MCC changed between 0.1 and 0.7). Regarding global performance, the combination of SMO with CDK FP showed the lowest IN/A training ratio (2:1–40:1) and the highest MCC.

## Supporting information

S1 Fig The panels show the dependency of machine learning-based virtual screening on the IN/A training size for all of the protein targets studied (panel A–CDK FP, and MACCS FP in B). The colored lines denote the type of evaluated parameter used (blue–recall, red–precision, magenta–MCC and green–PR plot).
(PDF)

S2 Fig The dependency of the IN/A training ratio on the cost-effectiveness thresholds for different screening library sizes obtained for all of the targets used. (PDF)

S3 Fig The dependency of the optimal IN/A training ratio from the size of the screening library, obtained for several arbitrarily selected cost-effectiveness thresholds for all of the targets studied. (PDF)

S1 Table The optimal IN/A training ratio obtained for all screening libraries using a cost-effectiveness cutoff = 0.03. (PDF)

S1 File Additional study performed to explain the dependency of the protein target on the performance of ML-based VS. The file contains the results and a discussion on the influence of the target type and screening library size on the performance of ML-based virtual screening.
(PDF)

S2 File The results of the two-way ANOVA. The file contains the interaction plots and analyses of variance (Tests of between-subjects effects table) for testing the significance of the main effect and interactions between them. The null hypothesis was no interaction between different effects on the global performance of virtual screening given by MCC, an alpha level was set at 0.0001.
(PDF)

S3 File A zip file containing datasets used and results obtained in this study. (RAR)
