# Supplementary material for: The influence of the negative-positive ratio and screening database size on the performance of machine learning-based virtual screening
Source: PLoS One. 2017 Apr 6;12(4):e0175410. doi: 10.1371/journal.pone.0175410 (PMC5383296; doi:10.1371/journal.pone.0175410)

HIV Pr (CDK FP)

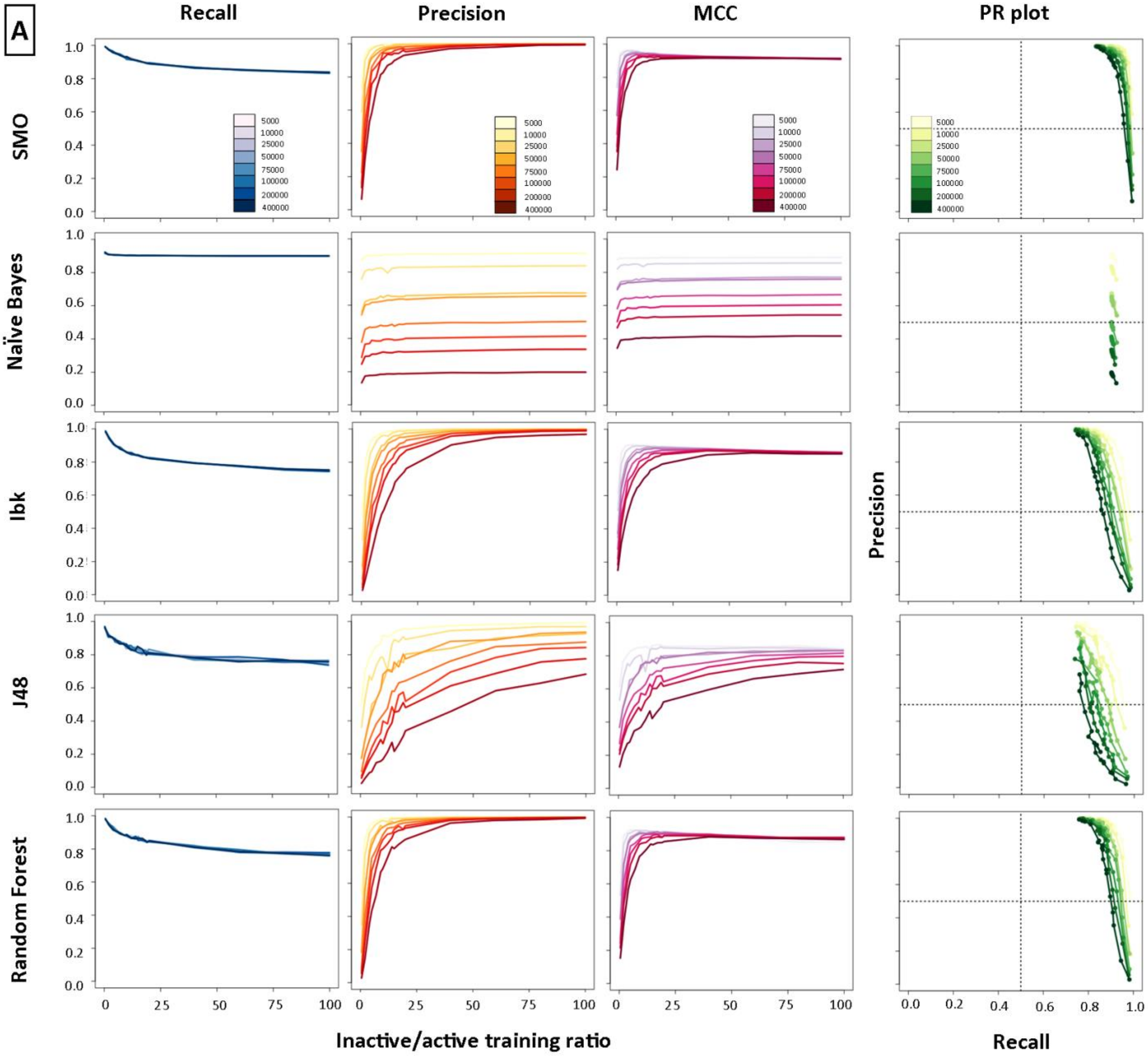
A
Recall
Precision
MCC
PR plot
SMO
Naïve Bayes
Ibk
J48
Random Forest
5000
10000
25000
50000
75000
100000
200000
400000
1.0
0.8
0.6
0.4
0.2
0.0
0
25
50
75
100
Inactive/active training ratio
Precision
Recall

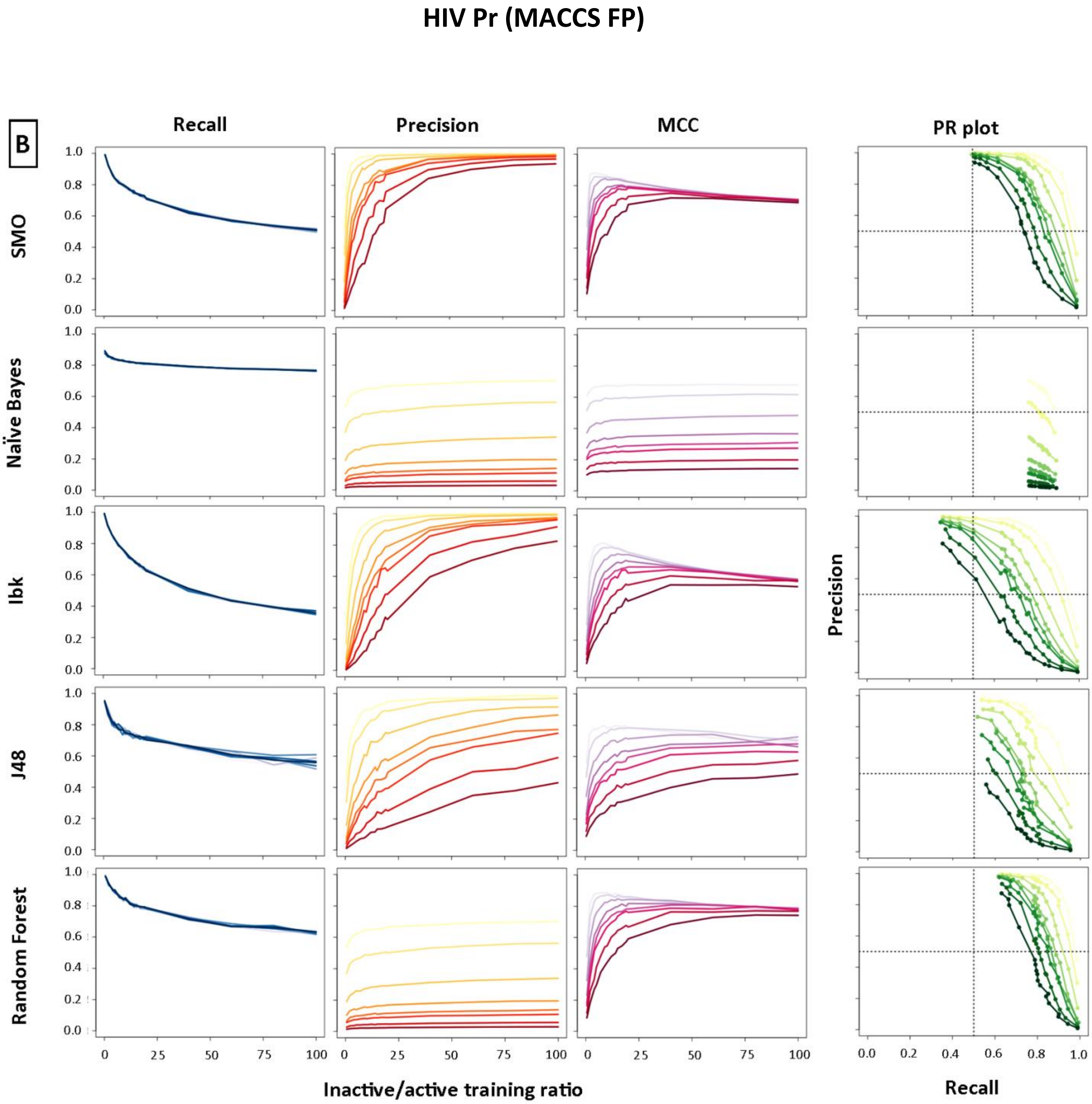

HIV Pr (MACCS FP)
B
Recall
Precision
MCC
PR plot
SMO
Naïve Bayes
Ibk
J48
Random Forest
Precision
Inactive/active training ratio
Recall
0.0
0.2
0.4
0.6
0.8
1.0
0
25
50
75
100

SERT (CDK FP)

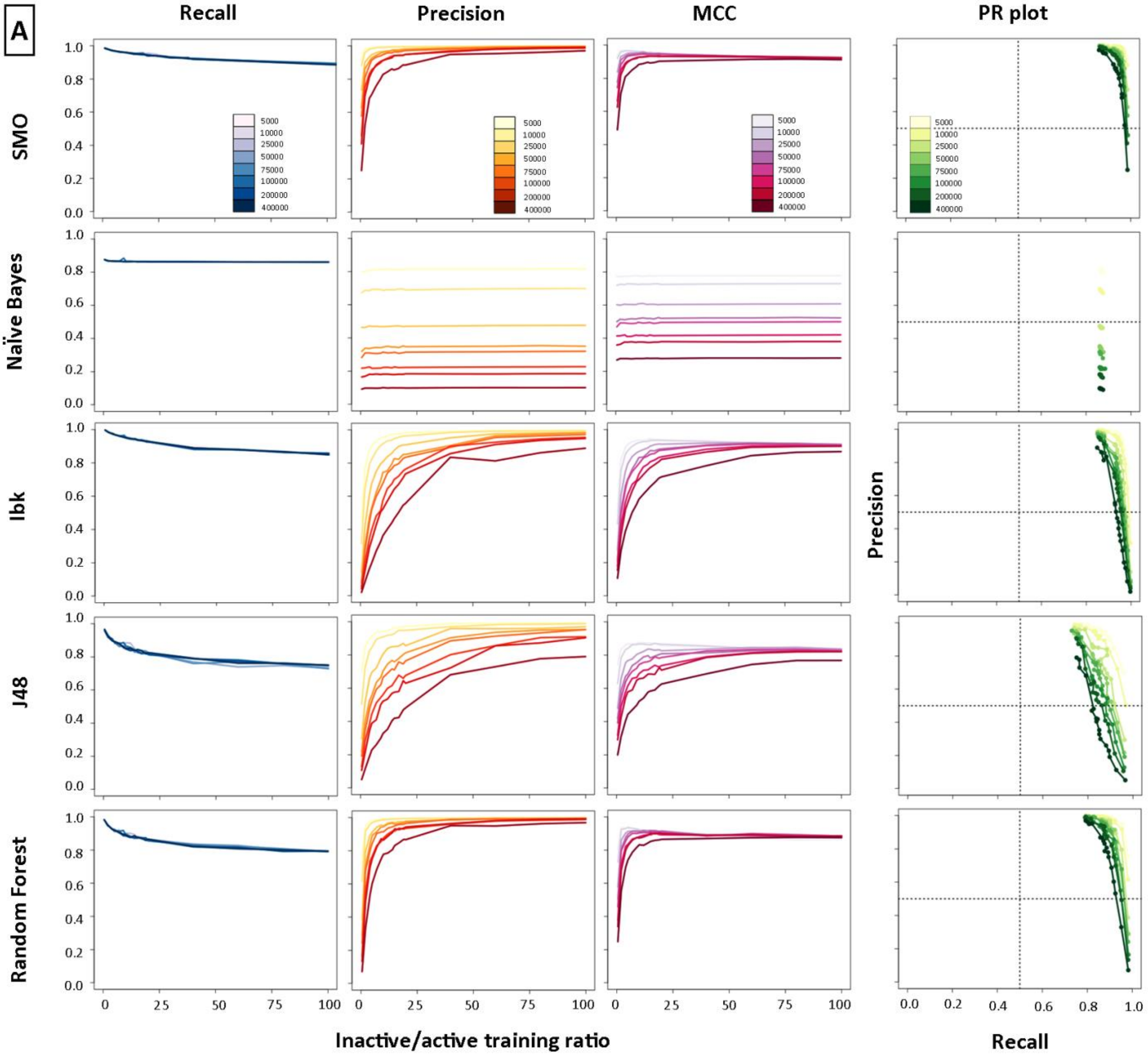
A
Recall
Precision
MCC
PR plot
SMO
Naïve Bayes
Ibk
J48
Random Forest
5000
10000
25000
50000
75000
100000
200000
400000
Inactive/active training ratio
Precision
Recall

SERT (MACCS FP)

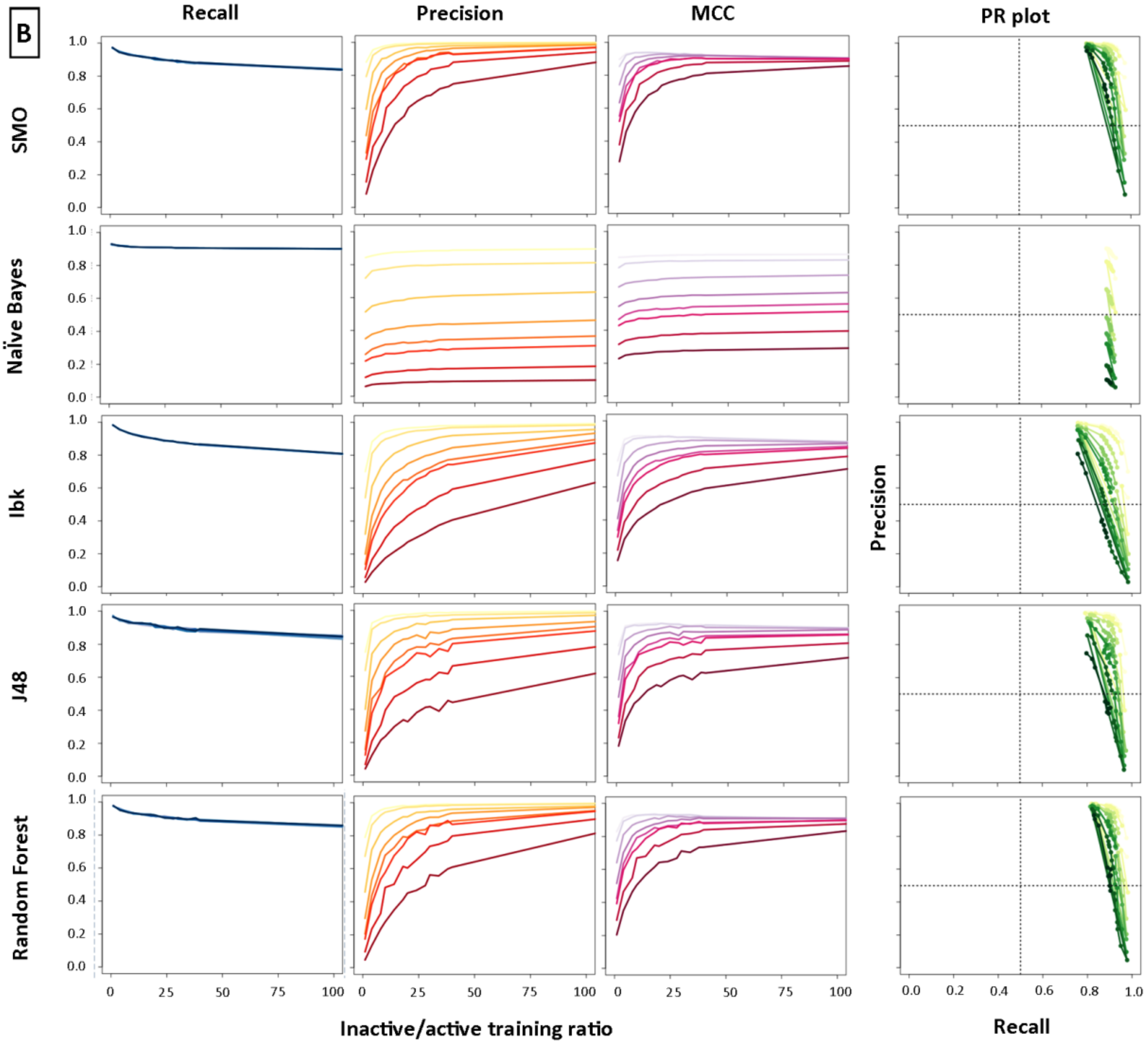

ER-α (CDK FP)

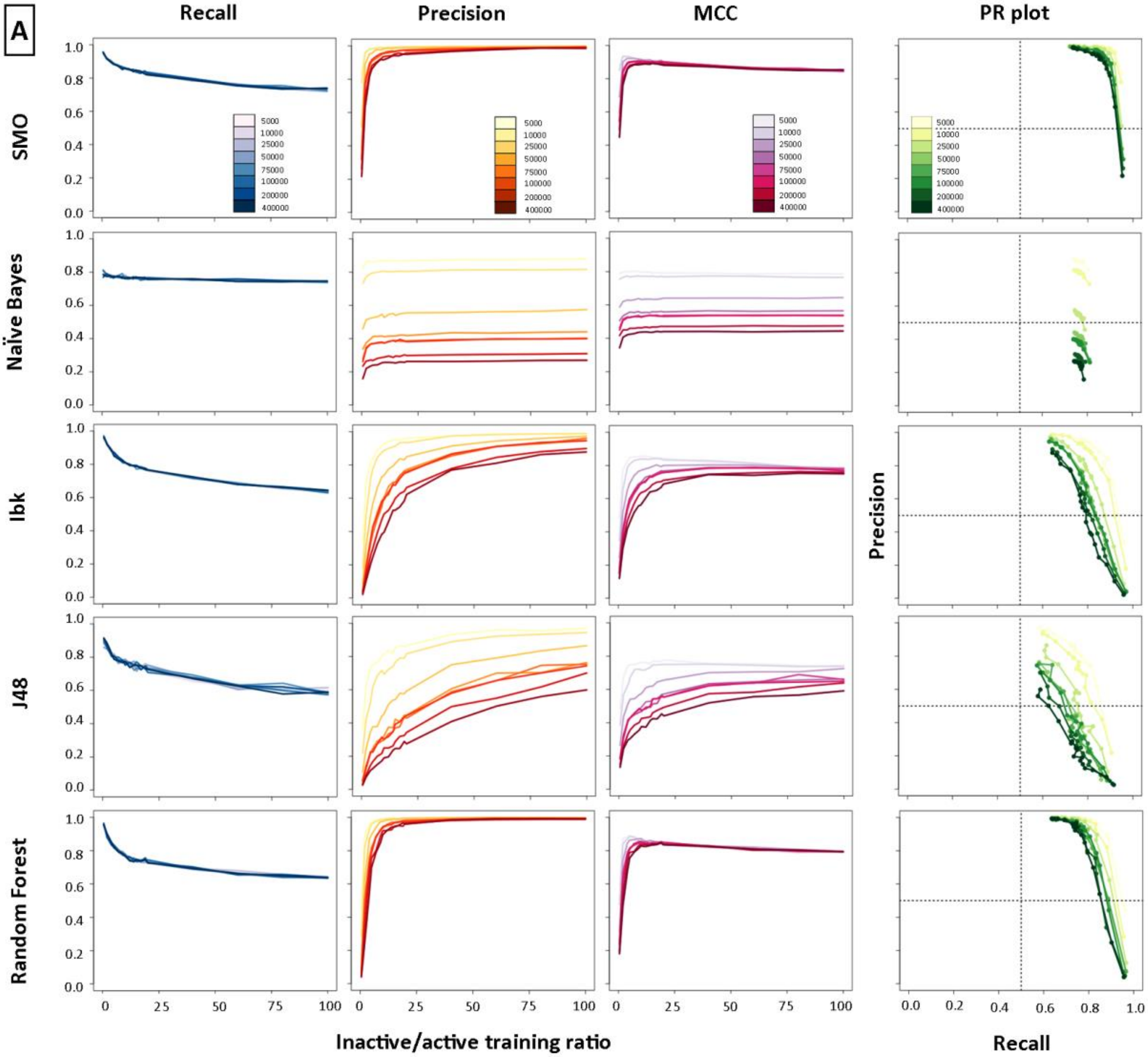
A
Recall
Precision
MCC
PR plot
SMO
Naïve Bayes
Ibk
J48
Random Forest
5000
10000
25000
50000
75000
100000
200000
400000
Precision
Inactive/active training ratio
Recall

ER-α (MACCS FP)

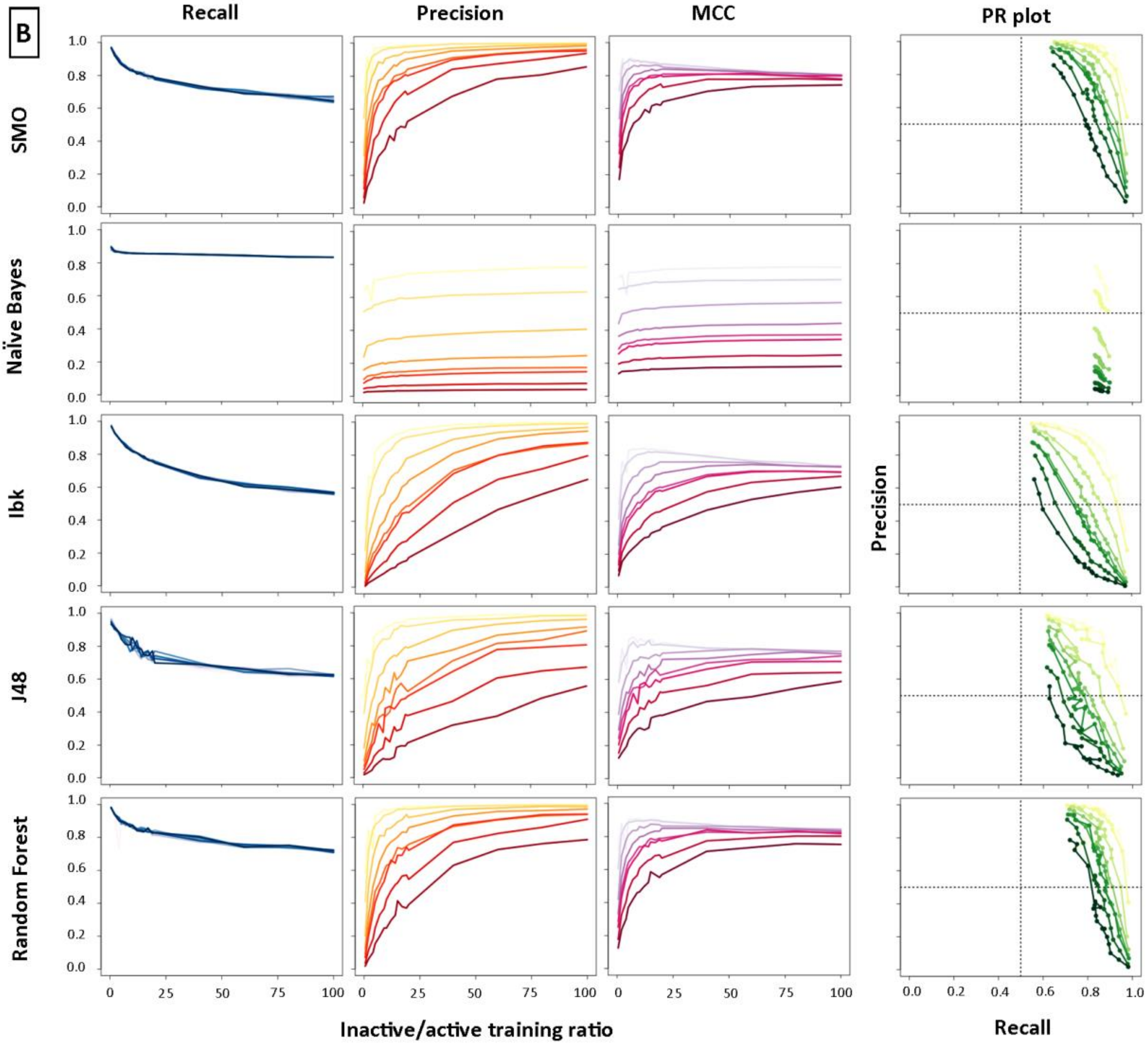
B
Recall
Precision
MCC
PR plot
SMO
Naïve Bayes
Ibk
J48
Random Forest
Precision
Inactive/active training ratio
Recall

AChE (CDK FP)

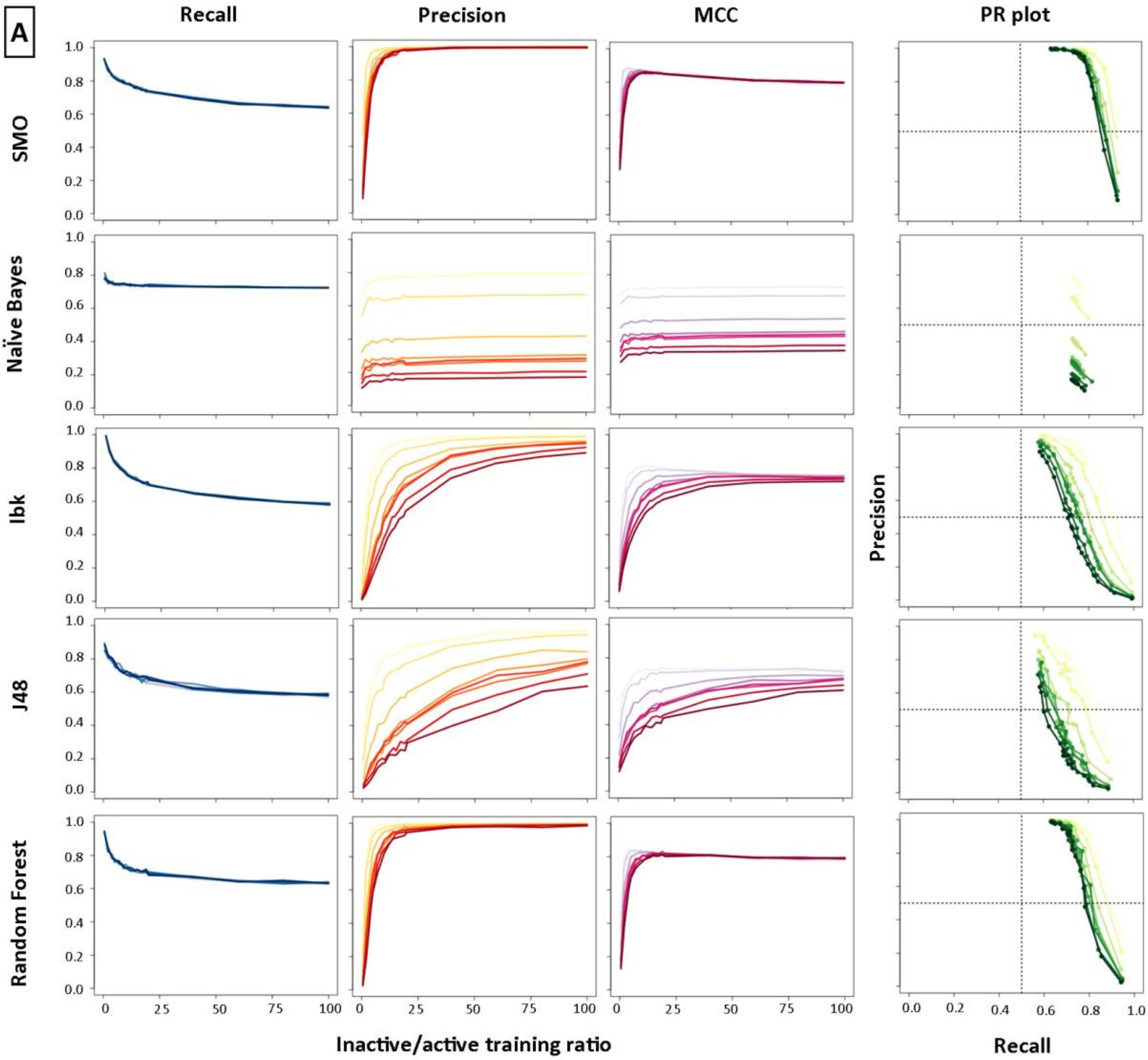

A
Recall
Precision
MCC
PR plot
SMO
Naïve Bayes
Ibk
J48
Random Forest
0.0
0.2
0.4
0.6
0.8
1.0
0
25
50
75
100
Inactive/active training ratio
Precision
Recall

AChE (MACCS FP)

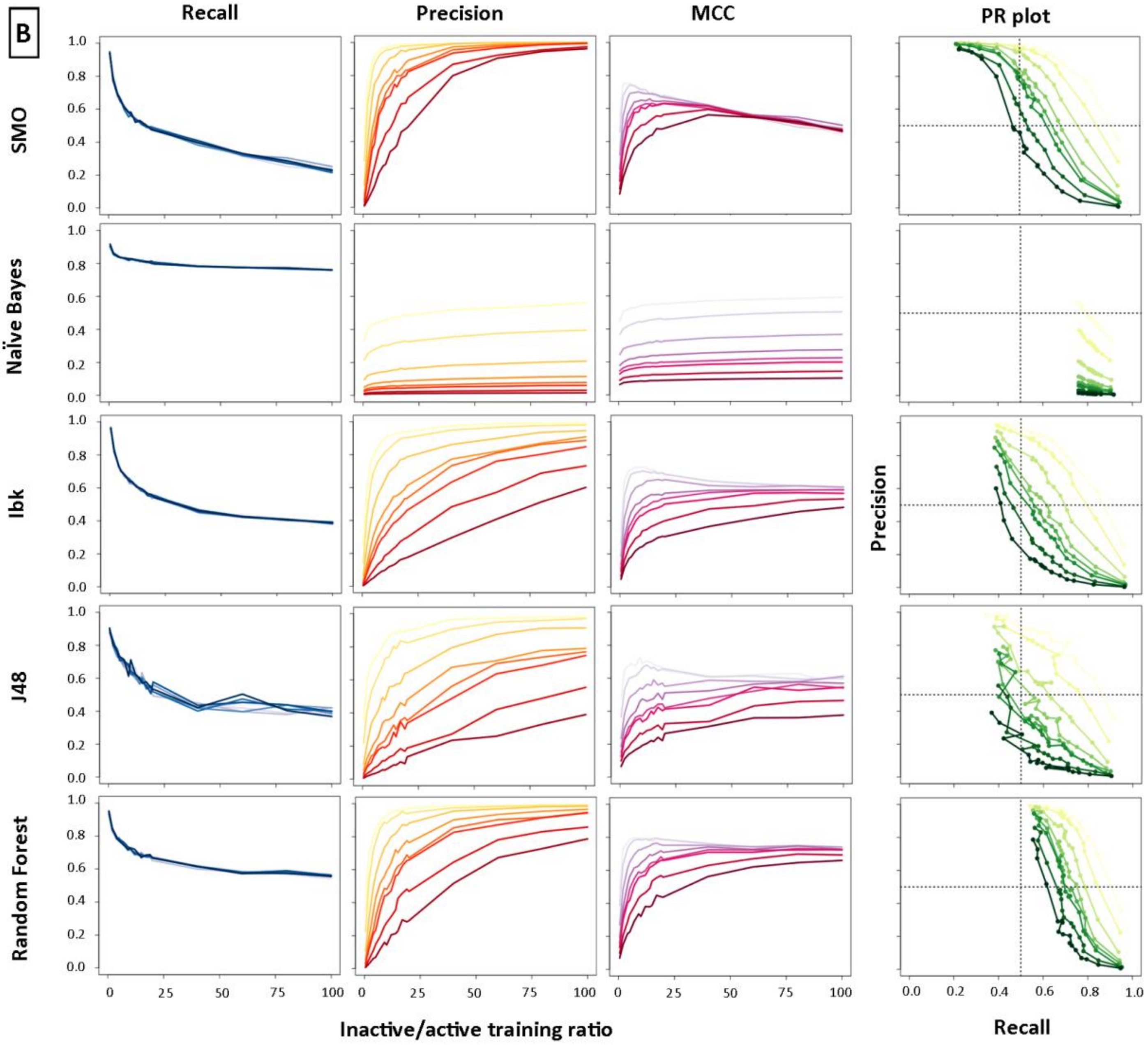

PDE5 (CDK FP)

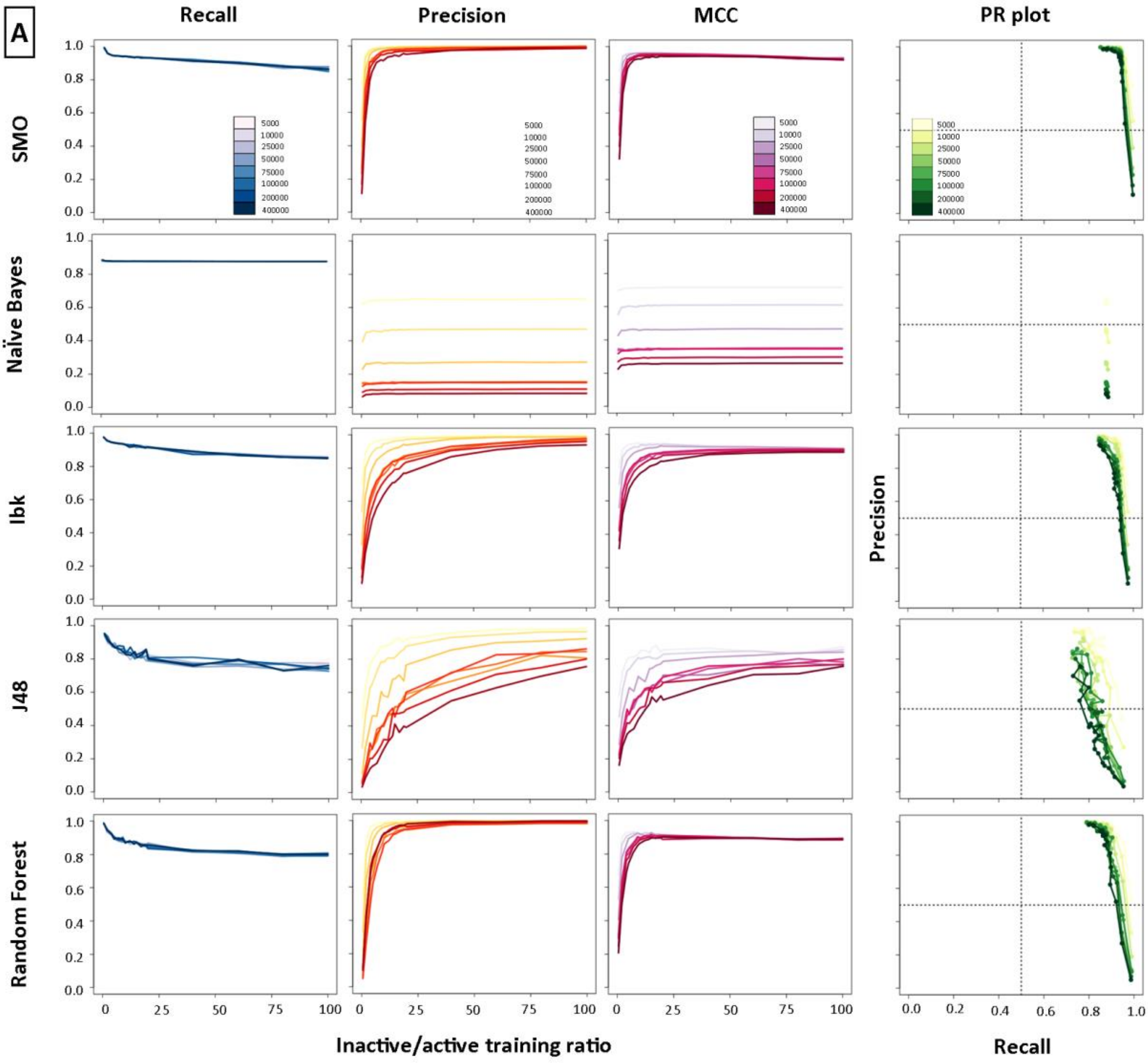

A
Recall
Precision
MCC
PR plot
SMO
Naïve Bayes
Ibk
J48
Random Forest
5000
10000
25000
50000
75000
100000
200000
400000
Inactive/active training ratio
Precision
Recall

PDE5 (MACCS FP)

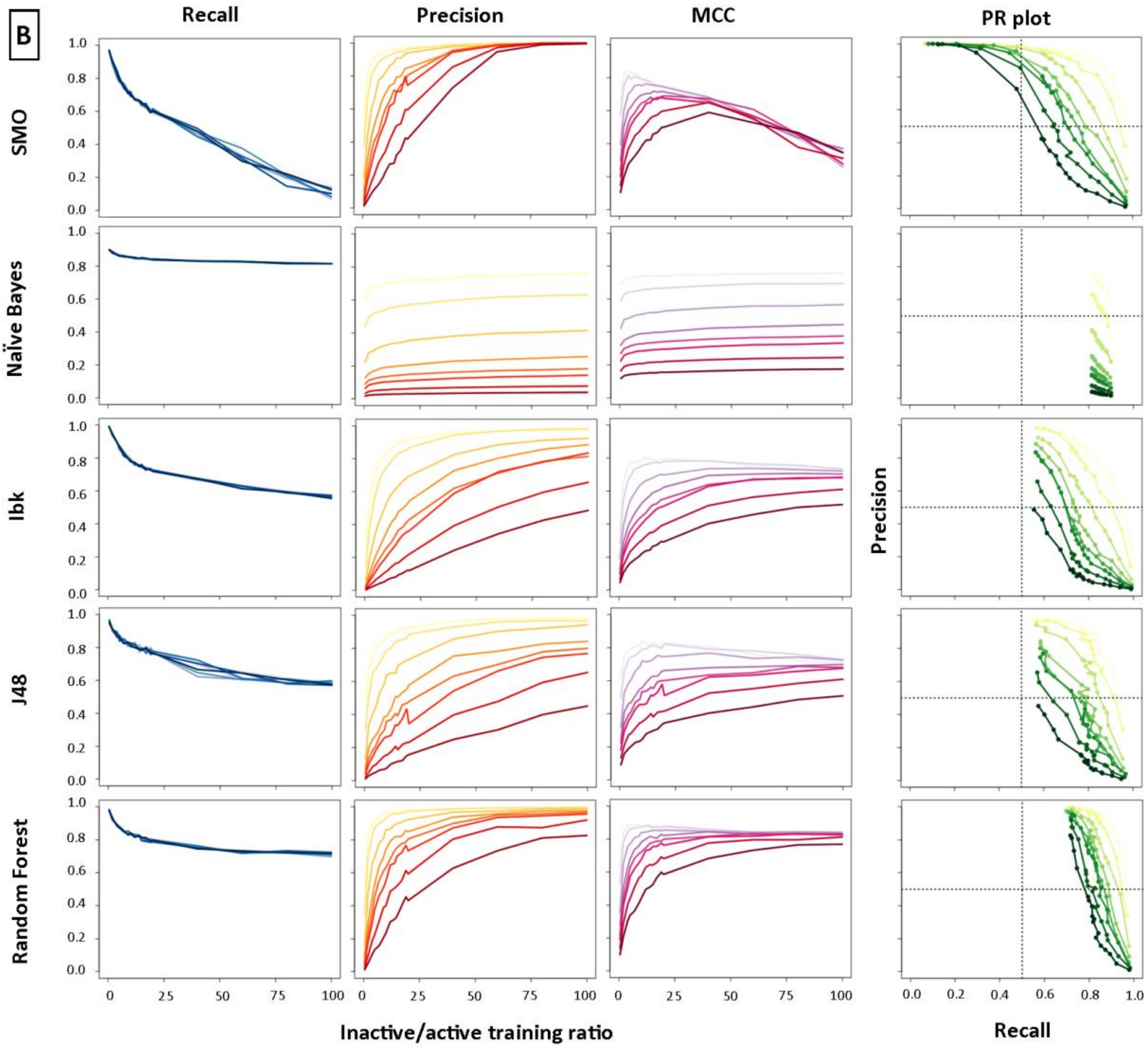

CDK2 (CDK FP)

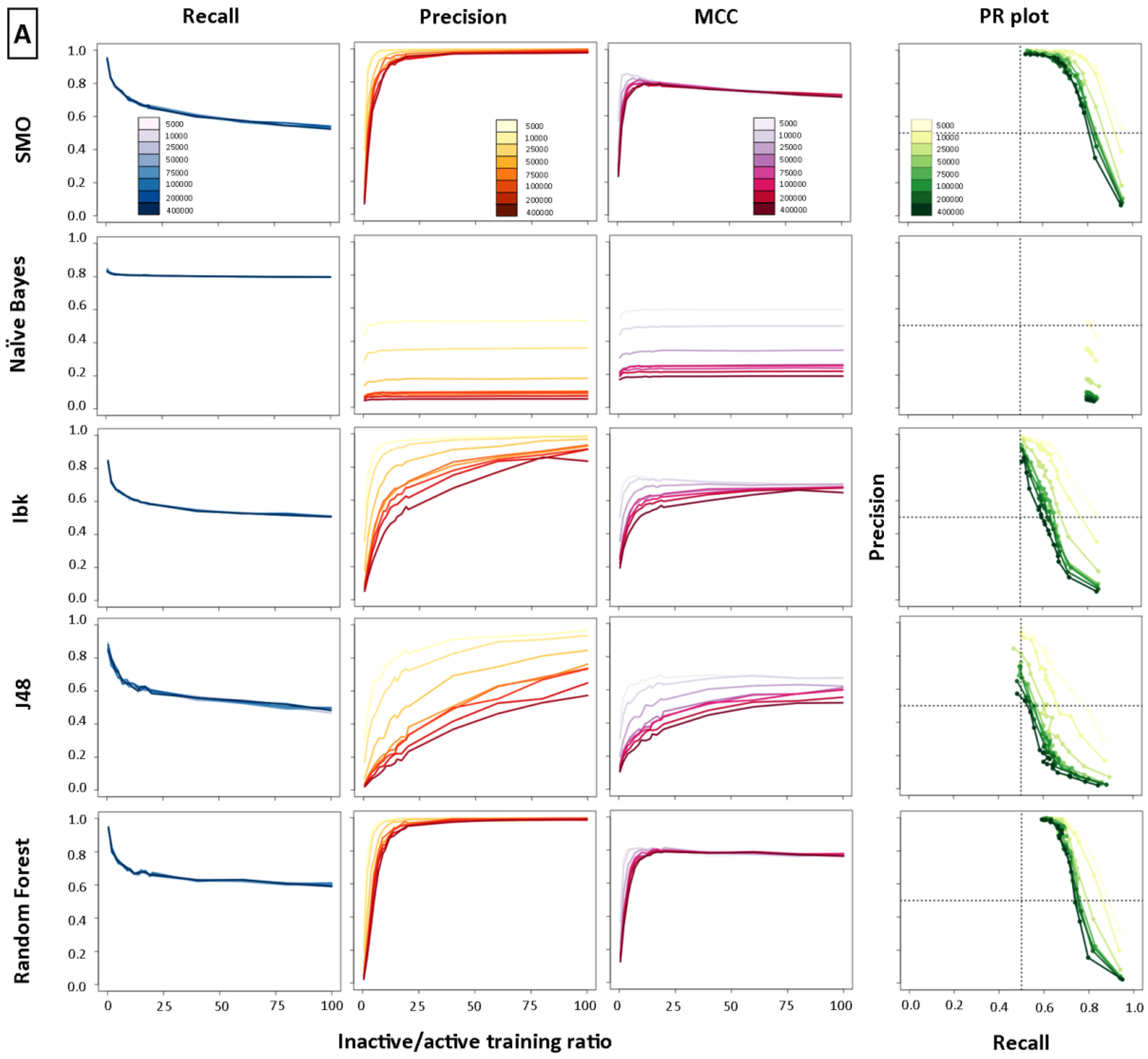
A
Recall
Precision
MCC
PR plot
SMO
Naïve Bayes
Ibk
J48
Random Forest
5000
10000
25000
50000
75000
100000
200000
400000
1.0
0.8
0.6
0.4
0.2
0.0
0
25
50
75
100
Inactive/active training ratio
Precision
Recall

CDK2 (MACCS FP)

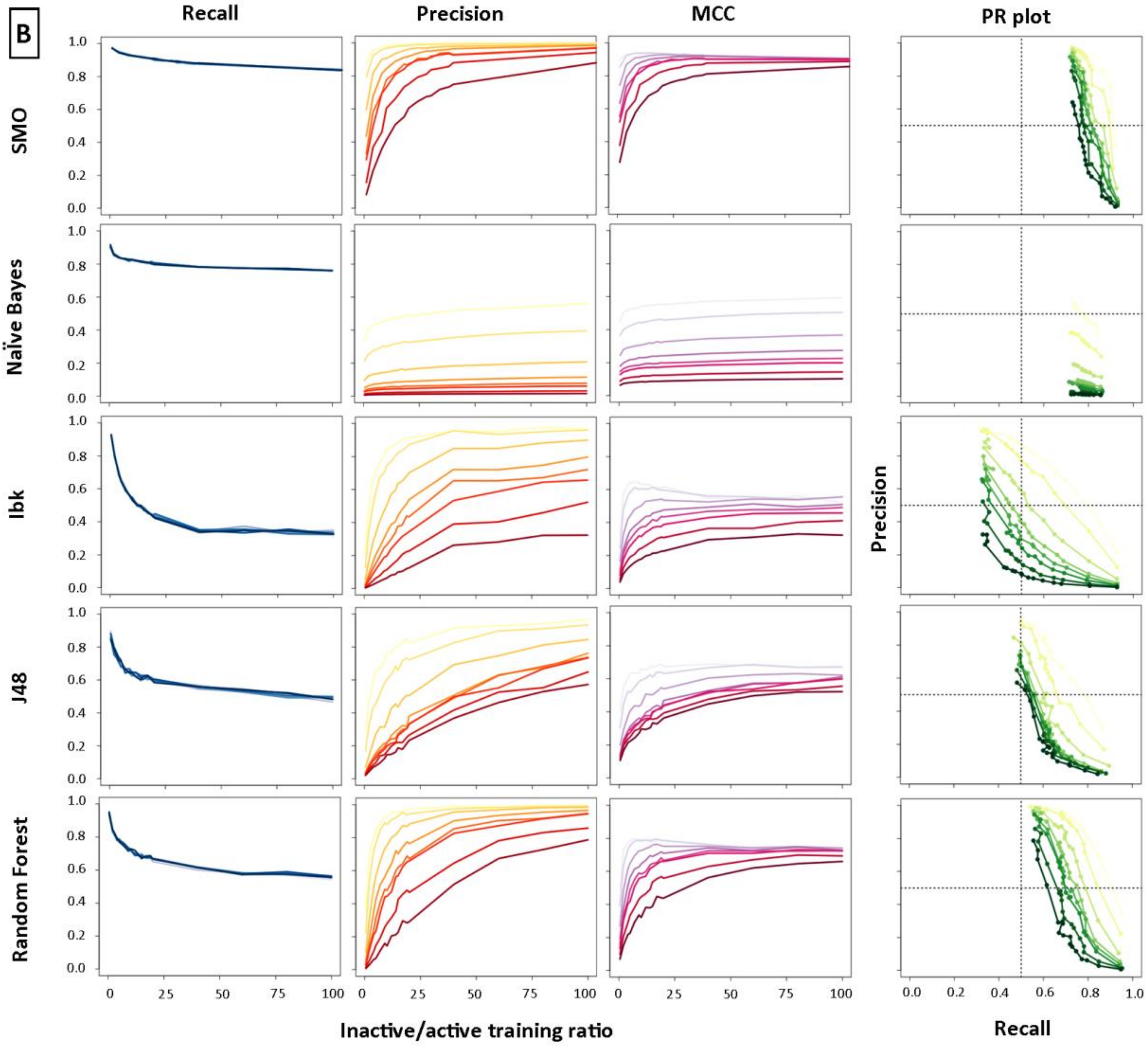

CRF1 (CDK FP)

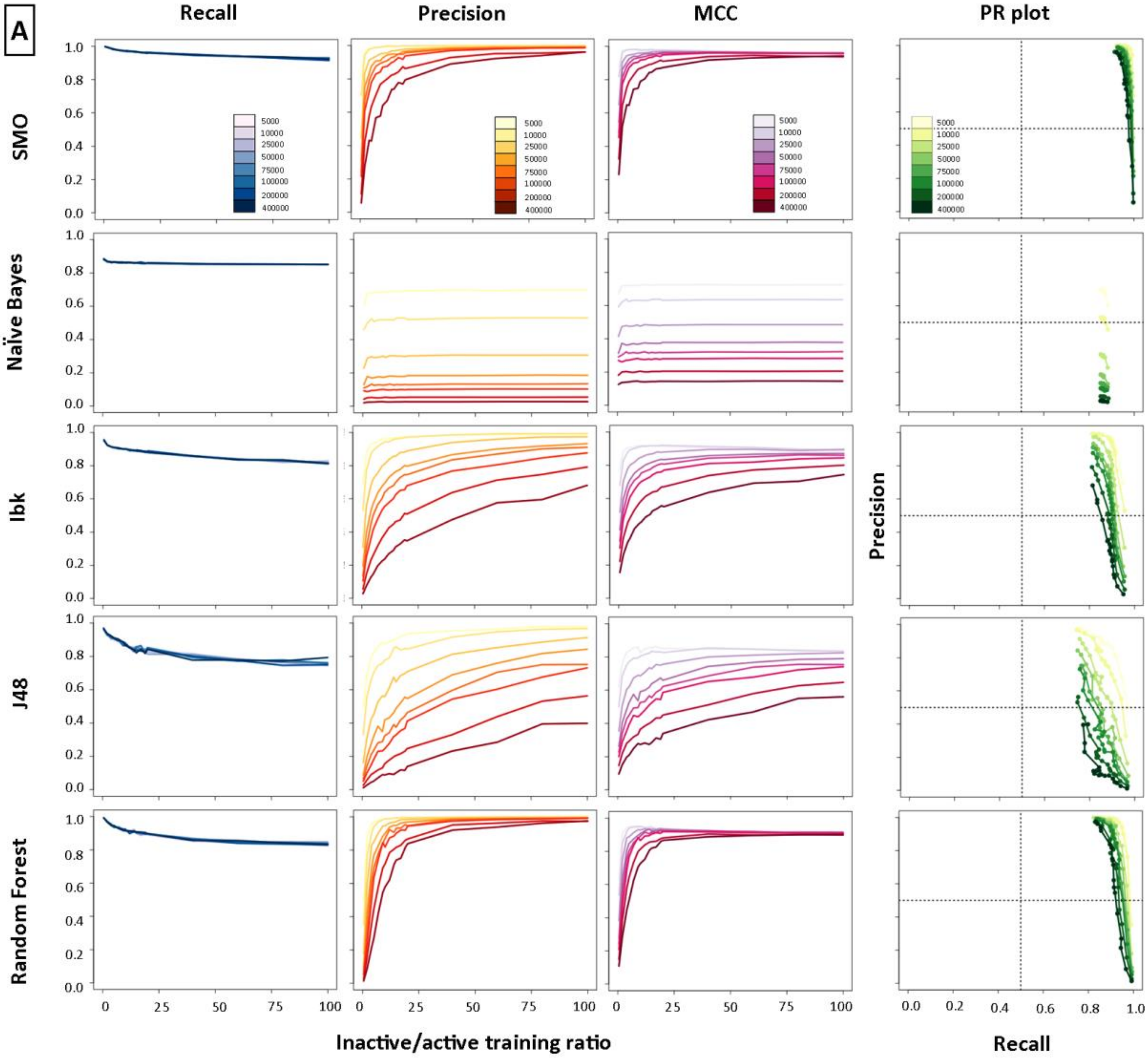
A
Recall
Precision
MCC
PR plot
SMO
Naïve Bayes
Ibk
J48
Random Forest
5000
10000
25000
50000
75000
100000
200000
400000
Inactive/active training ratio
Precision
Recall

CRF1 (MACCS FP)

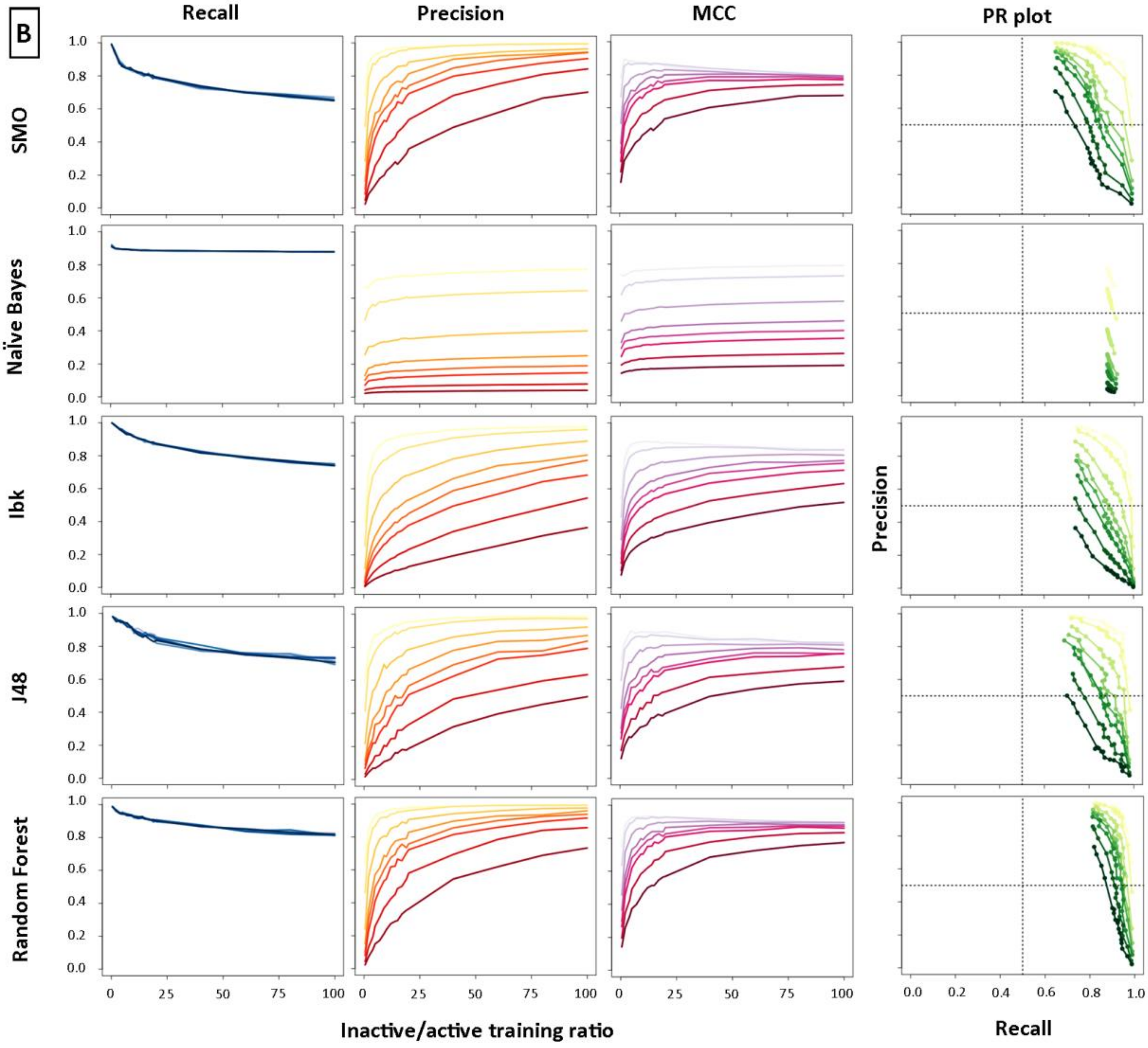

Supplement: S1 Fig — The colored lines denote the type of evaluated parameter used (blue–recall, red–precision, magenta–MCC and green–PR plot). (PDF) [file pone.0175410.s001.pdf]
