# Supplementary figures and images for: The influence of the negative-positive ratio and screening database size on the performance of machine learning-based virtual screening
Source: PLoS One. 2017 Apr 6;12(4):e0175410. doi: 10.1371/journal.pone.0175410 (PMC5383296; doi:10.1371/journal.pone.0175410)

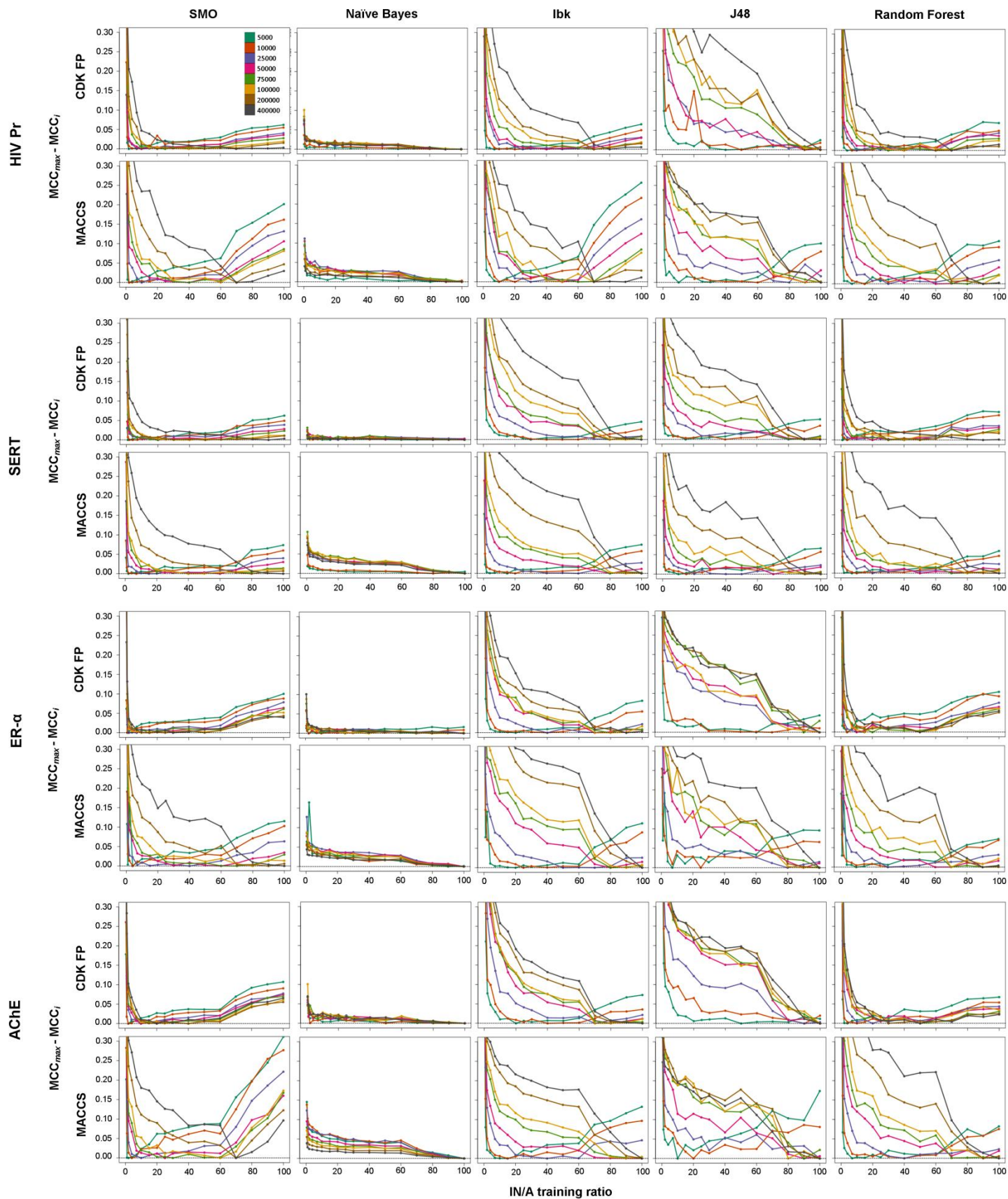

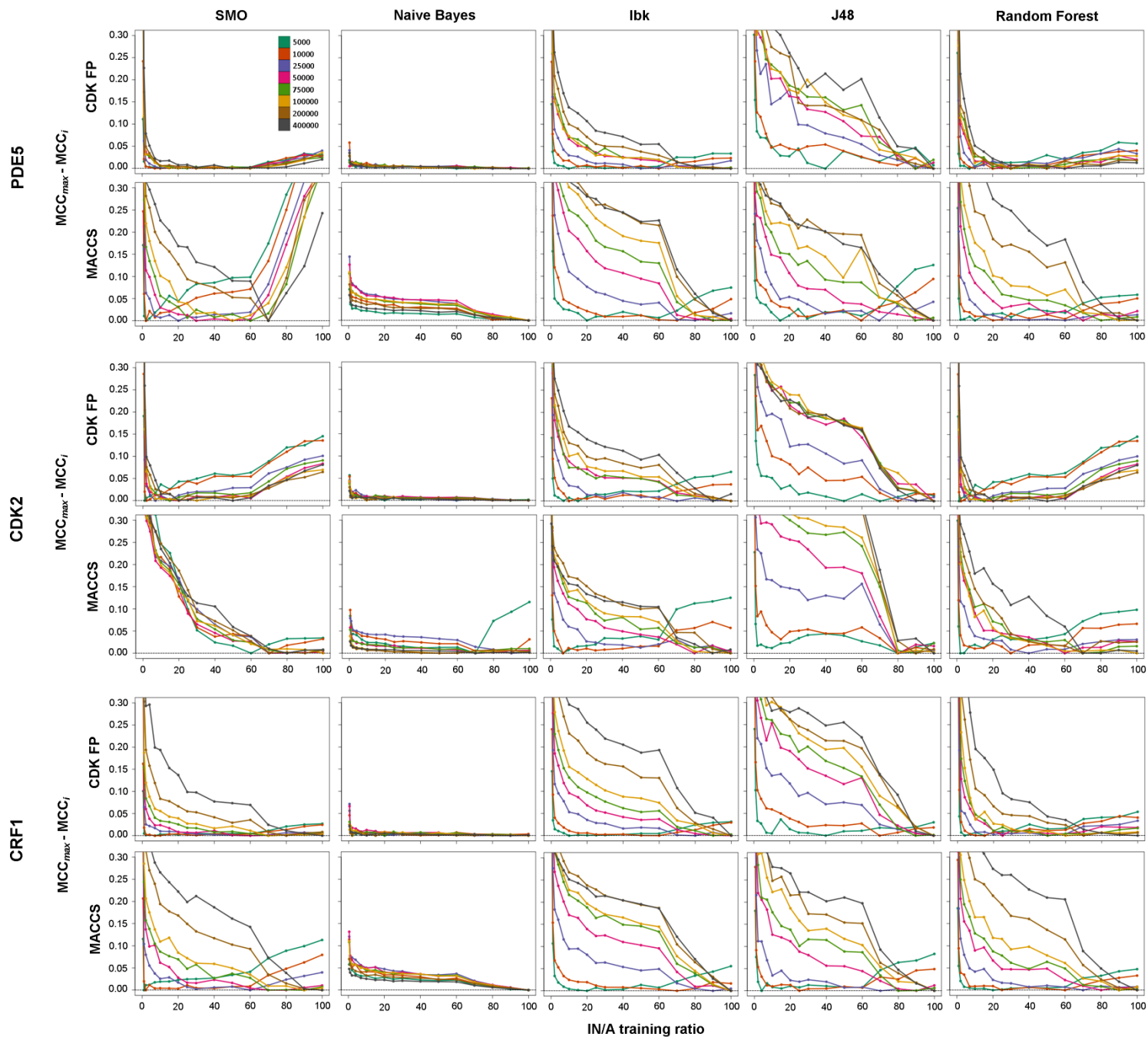

Supplement: S2 Fig — (PDF) [file pone.0175410.s002.pdf]
