# Supplementary material for: The influence of the negative-positive ratio and screening database size on the performance of machine learning-based virtual screening
Source: PLoS One. 2017 Apr 6;12(4):e0175410. doi: 10.1371/journal.pone.0175410 (PMC5383296; doi:10.1371/journal.pone.0175410)
Supplement: S3 Fig — (PDF) [file pone.0175410.s003.pdf]

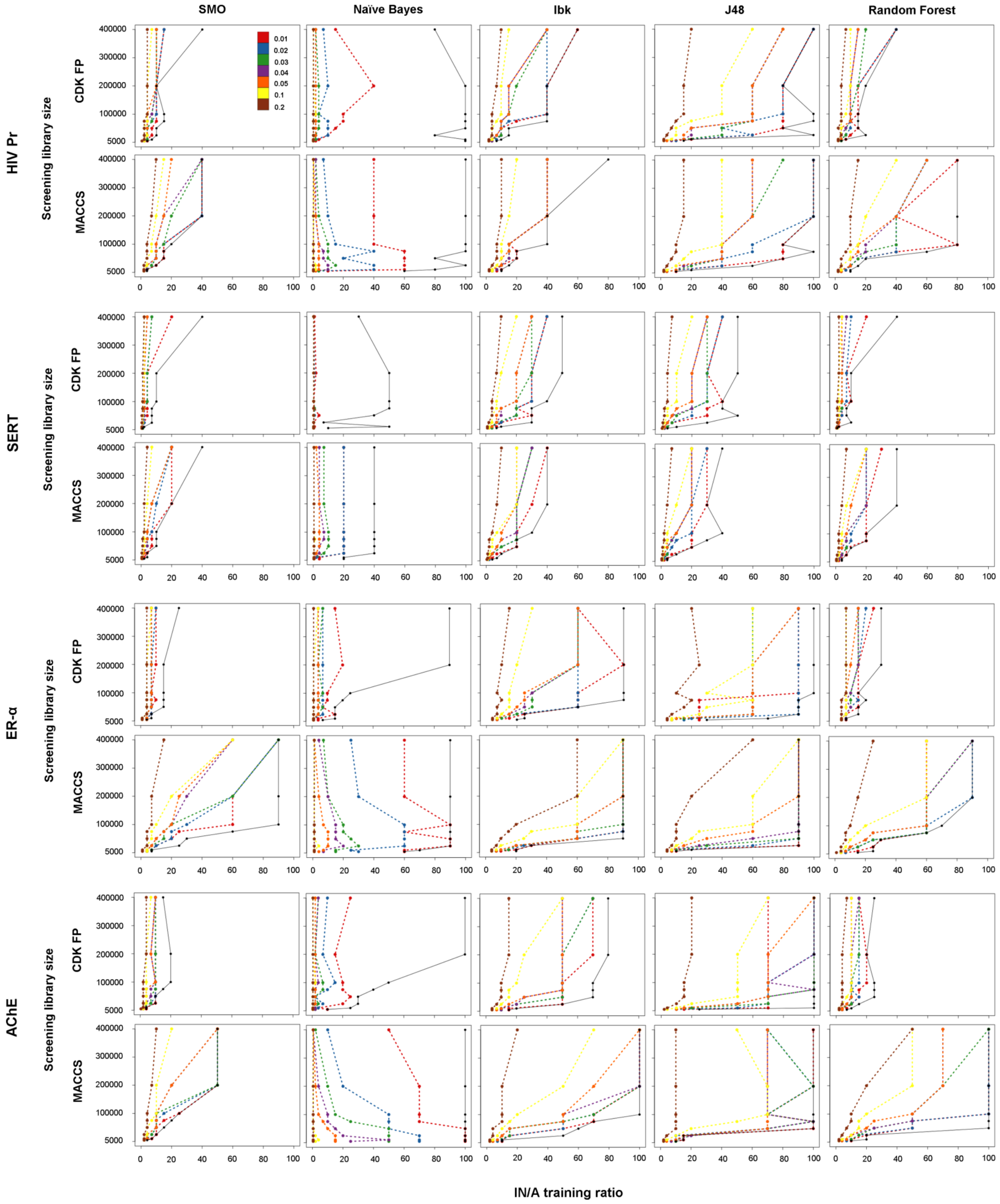
SMO
Naïve Bayes
Ibk
J48
Random Forest
0.01
0.02
0.03
0.04
0.05
0.1
0.2
HIV Pr
SERT
ER-α
AChE
Screening library size
CDK FP
MACCS
IN/A training ratio

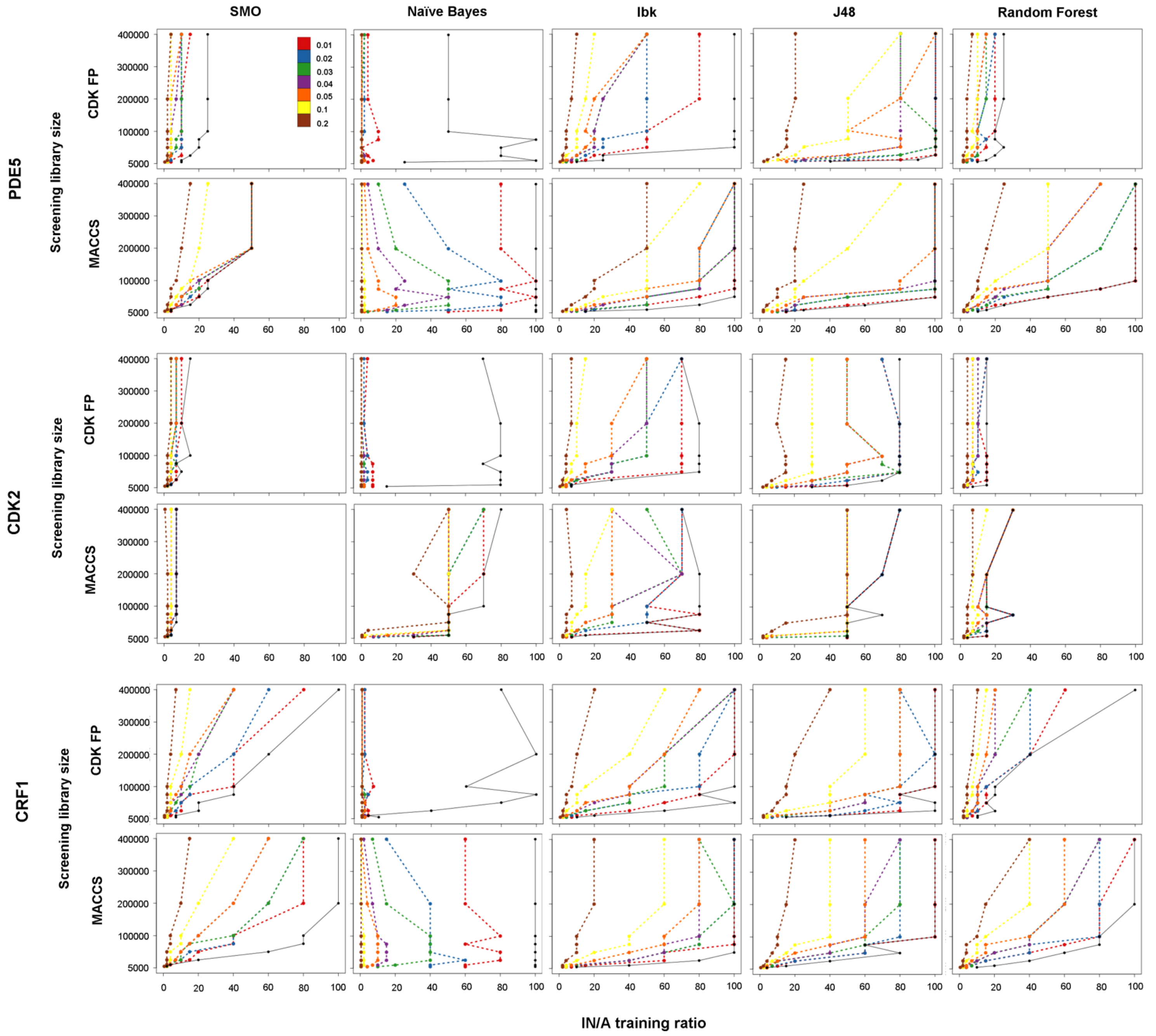
SMO
Naïve Bayes
Ibk
J48
Random Forest
0.01
0.02
0.03
0.04
0.05
0.1
0.2
PDE5
CDK2
CRF1
Screening library size
CDK FP
MACCS
IN/A training ratio
