# Supplementary material for: The influence of the negative-positive ratio and screening database size on the performance of machine learning-based virtual screening
Source: PLoS One. 2017 Apr 6;12(4):e0175410. doi: 10.1371/journal.pone.0175410 (PMC5383296; doi:10.1371/journal.pone.0175410)
Supplement: S1 Table — (PDF) [file pone.0175410.s004.pdf]

S1 Table. The optimal IN/A training ratio obtained for cost-effectiveness cutoff = 0.03.

Target	Screening library size	Best IN/A training ratio									
		SMO		NB		Ibk		J48		RF	
		CDK FP	MACCS	CDK FP	MACCS	CDK FP	MACCS	CDK FP	MACCS	CDK FP	MACCS
$5\text{-}HT_{1A}R$	5000	2	2	2	60	10	10	10	2	4	4
	10000	2	2	0.5	60	15	20	15	4	4	7
	25000	4	4	0.5	60	40	60	60	10	7	10
	50000	7	7	0.5	40	60	80	60	15	10	15
	75000	10	10	2	40	80	80	80	40	15	40
	100000	15	10	0.5	40	80	80	60	60	15	40
	200000	40	20	0.5	15	100	100	80	40	15	60
	400000	40	40	0.5	10	100	100	80	80	40	60
HIV Pr	5000	2	2	0.5	4	4	4	7	4	4	4
	10000	4	4	2	10	4	7	15	10	4	4
	25000	4	7	2	15	10	10	40	20	7	10
	50000	4	10	4	10	10	15	40	40	7	15
	75000	7	10	4	10	10	15	60	40	10	20
	100000	7	15	4	10	15	15	60	40	10	40
	200000	10	20	4	4	20	40	60	60	15	40
	400000	10	40	2	2	40	40	80	80	20	60
SERT	5000	1	1	0.5	0.5	4	1	2	1	1	1
	10000	1	1	0.5	1	4	2	4	1	1	2
	25000	1	2	0.5	7	7	4	7	4	2	4
	50000	2	4	0.5	10	20	10	10	4	2	7
	75000	2	4	1	10	20	20	20	7	4	7
	100000	4	4	0.5	10	20	20	30	10	4	7
	200000	4	7	0.5	7	30	20	30	20	4	20
	400000	7	20	0.5	7	30	30	30	20	7	20
ER-α	5000	4	4	1	15	7	7	7	7	4	4
	10000	4	7	1	15	10	15	15	10	7	7
	25000	4	7	4	30	20	20	60	30	7	15
	50000	7	15	7	25	30	60	60	90	10	25
	75000	7	15	4	20	30	60	60	90	10	60
	100000	7	20	7	20	30	90	60	90	10	60
	200000	7	60	4	10	60	90	60	90	15	60
	400000	7	90	7	7	60	90	60	90	15	90
AChE	5000	2	2	2	50	10	4	10	10	4	4
	10000	2	4	2	50	10	7	20	10	4	10
	25000	7	7	4	50	20	15	70	20	10	15
	50000	7	10	2	50	50	15	70	70	10	25
	75000	10	10	7	25	50	50	100	100	10	50
	100000	10	10	7	15	50	70	100	70	10	50
	200000	10	50	2	10	50	100	100	100	15	70
	400000	10	50	4	2	70	100	100	70	15	100
PDE5	5000	2	2	0.5	4	4	10	15	10	4	4
	10000	2	4	2	20	10	15	25	15	4	7

	25000	4	10	2	50	10	50	80	20	10	15
	50000	7	10	0.5	50	20	50	100	50	10	20
	75000	7	20	0.5	50	20	80	100	100	10	50
	100000	10	20	0.5	50	20	80	100	100	10	50
	200000	10	50	0.5	20	25	100	80	100	15	80
	400000	10	50	2	10	50	100	80	100	15	100
CDK2	5000	2	15	2	7	4	4	7	4	4	4
	10000	2	30	2	10	4	4	30	50	4	4
	25000	4	30	2	15	10	10	30	50	7	10
	50000	4	15	2	4	30	30	80	50	7	15
	75000	7	15	2	4	30	30	70	50	7	15
	100000	4	15	2	2	50	30	70	50	10	15
	200000	7	15	0.5	0.5	50	70	50	50	10	15
	400000	7	30	0.5	0.5	50	50	50	50	10	30
CRF1	5000	2	2	2	10	4	4	4	4	4	4
	10000	2	2	2	20	4	10	15	7	4	7
	25000	4	10	2	40	15	40	40	10	7	10
	50000	7	10	2	40	40	60	60	40	10	20
	75000	10	15	2	40	40	80	60	60	10	40
	100000	15	40	0.5	40	60	80	80	60	10	40
	200000	20	60	0.5	15	60	100	80	80	20	60
	400000	40	80	0.5	7	100	80	80	80	40	80
