# Supplementary material for: The influence of the negative-positive ratio and screening database size on the performance of machine learning-based virtual screening
Source: PLoS One. 2017 Apr 6;12(4):e0175410. doi: 10.1371/journal.pone.0175410 (PMC5383296; doi:10.1371/journal.pone.0175410)
Supplement: S1 File — The file contains the results and a discussion on the influence of the target type and screening library size on the performance of ML-based virtual screening. (PDF) [file pone.0175410.s005.pdf]

Study of the influence of the type of protein target to performance of ML-based virtual screening

The average pairwise similarity (using CDK FP and Tanimoto metric) was calculated between all targets and showed (Fig 1) high structural diversity (Tanimoto coefficient ranged from 0.21 to 0.36 between different targets, and 0.27 to 0.43 inside targets). The most structurally distant ligands was found for SERT, whereas HIV Pr and PDE5 showed the lowest diversity among all targets used. Interestingly, the HIV Pr and PDE5 ligands had highest internal similarity (Tc equal 0.39 and 0.43, respectively), which is related with the low chemotype diversity.

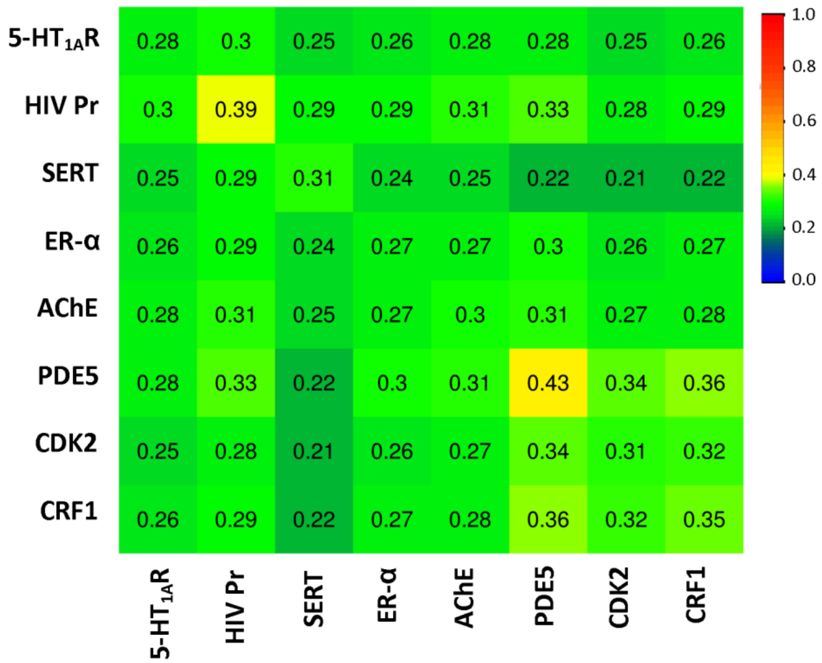

Fig 1. The heat map showing inter- and intra-target average Tc pairwise similarity calculated using Tanimoto metric and CDK FP.

As the same screening libraries and structurally different ligands were used in virtual screening experiments, the pairwise similarity calculations (using CDK FP and Tanimoto metric) were performed. For each protein target, the violin plot (Fig 2) shows density of the screening compounds with high similarity to active compounds (Tanimoto coefficient higher than 0.7) was plotted. The results revealed that the number of compounds found in screening libraries, which are highly similar to actives depends on the target type and size of screening library.

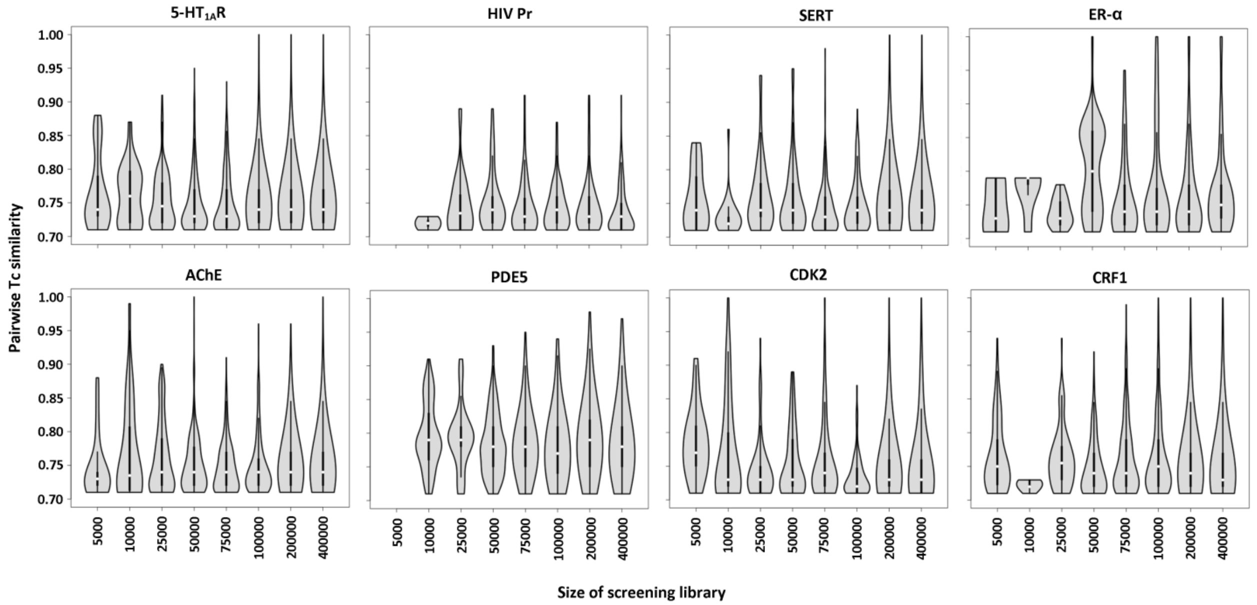

Fig 2. Violin plots showing density of the screening compounds with high structural similarity to active compounds (Tanimoto coefficient higher than 0.7) for all targets studied.

However, no clear correlation between increasing of screening library size and the number of highly similar compounds (Tc higher than 0.9) to target ligands was observed. Globally, the 5-HT_{1A}R, AChE, CDK2 and CRF1 showed the highest, whereas HIV Pr the lowest density of highly similar to active compounds in screening libraries. Interestingly, the performed similarity analysis can be used to support the study of target influence on the ML-based VS performance.

As an example, we take the results obtained for combination of 100 k library, SMO, CDK FP and IN/A training ratio = 10:1 (Table 1).

Table 1. SMO performance metrics obtained for 100 k screening library size and IN/A training ratio 10:1.

Target	Recall	Precision	MCC
5-HT$_{1A}$R	0.80	0.66	0.73
HIV Pr	0.93	0.90	0.91
SERT	0.96	0.90	0.92
ER-α	0.86	0.95	0.91
AChE	0.79	0.93	0.85
PDE5	0.94	0.96	0.95
CDK2	0.72	0.88	0.79
CRF1	0.97	0.84	0.90

The targets having the lowest VS performance parameters (5-HT$_{1A}$R, AChE and CDK2) showed both the highest density of similar to active compounds and high internal chemotype diversity (Figure 1). Moreover, targets performed the best in VS experiments (HIV Pr and PDE5) showed both the least density of similar to active compounds and low internal chemotype diversity. However, for some targets there were no clear interplay between density of similar to active and internal diversity of target and obtained performance parameters. SERT showed very good performance, but simultaneously had noteworthy level of similar to active compounds (interestingly, not for considered library = 100 k) and medium ligand diversity. The next example - CRF1 showed good recall and MCC values, which might be explain by relatively low

chemotype diversity, and weaker precision, which might be influenced by slightly high density of similar to active compounds. An exception is ER-α, which showed good performance, but concurrently have the least diverse ligands and many similar to active compounds in screening libraries.

The discussed above tendency of precision to achieves the constant value and its significantly weaker dependency of the size of screening library might be connected with diversity and average similarity of screening compounds to actives as well. The results (Fig 2) depicts that for targets, where precision reaches maximum value rapidly, the active compounds are globally less similar to compounds from screening database, however the average similarity increases with database enlargement. When less compounds included in screening libraries are similar to actives, the less incorrectly classified negatives (FP) can be obtained.
