# Supplementary material for: The influence of the negative-positive ratio and screening database size on the performance of machine learning-based virtual screening
Source: PLoS One. 2017 Apr 6;12(4):e0175410. doi: 10.1371/journal.pone.0175410 (PMC5383296; doi:10.1371/journal.pone.0175410)
Supplement: S2 File — The file contains the interaction plots and analyses of variance (Tests of between-subjects effects table) for testing the significance of the main effect and interactions between them. The null hypothesis was no interaction between different effects on the global performance of virtual screening given by MCC, an alpha level was set at 0.0001. (PDF) [file pone.0175410.s006.pdf]

Two-way ANOVA

Case 1. Testing the effect of the ML method on the virtual screening performance given by MCC value for different protein targets used.

Interaction plot:

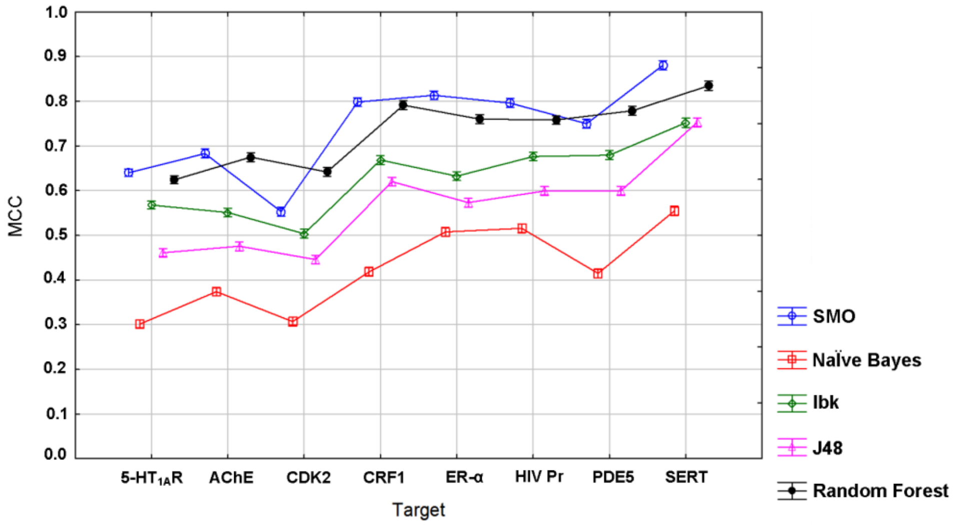

Analysis of Variance:

Source	SS	df	MS	F	p
Target	382.55	7	54.65	1595.8	0.0000
ML	748.23	4	187.06	5462.0	0.0000
Target*ML	35.29	28	1.26	36.8	0.0000
Error	1890.76	55210	0.03		

Case 2. Testing the effect of the molecular fingerprint on the virtual screening performance given by MCC value for different protein targets used.

Interaction plot:

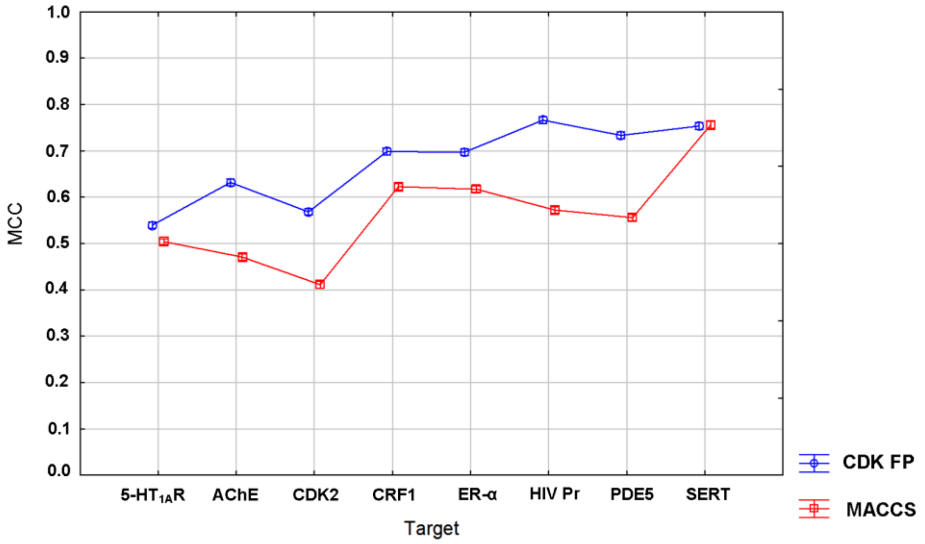

Analysis of Variance:

Source	SS	df	MS	F	p
Target	379.16	7	54.17	1214.3	0.0000
FP	164.33	1	164.33	3684.0	0.0000
Target*FP	62.44	7	8.92	200.0	0.0000
Error	2463.74	55234	0.04		

Case 3. Testing the effect of the molecular fingerprint on the virtual screening performance given by MCC value for different machine learning methods (ML) used.

Interaction plot:

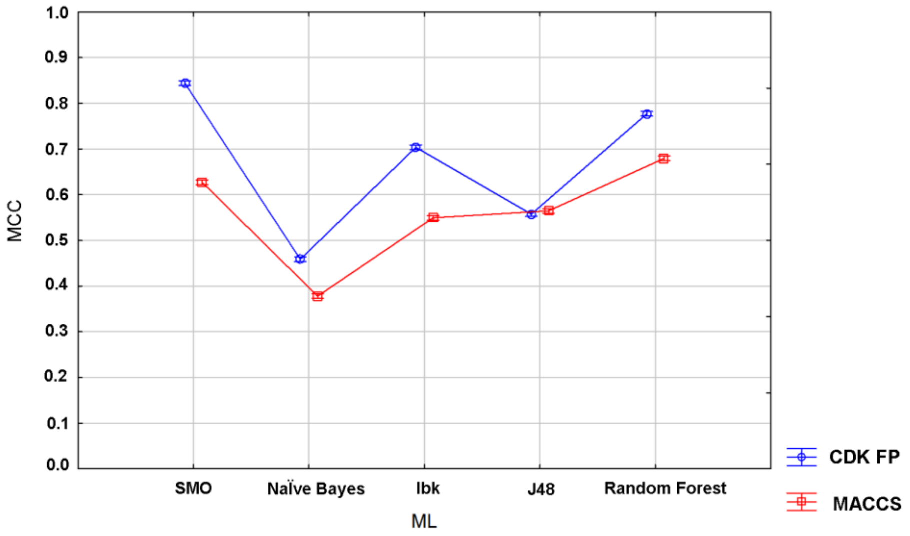

Analysis of Variance:

Source	SS	df	MS	F	p
FP	162.26	1	162.26	4335.2	0.0000
ML	765.76	4	191.44	5114.9	0.0000
FP*ML	77.41	4	19.35	517.0	0.0000
Error	2067.52	55240	0.04		

Case 4. Testing the effect of the molecular fingerprint type on the virtual screening performance given by MCC value for different screening library size used.

Interaction plot:

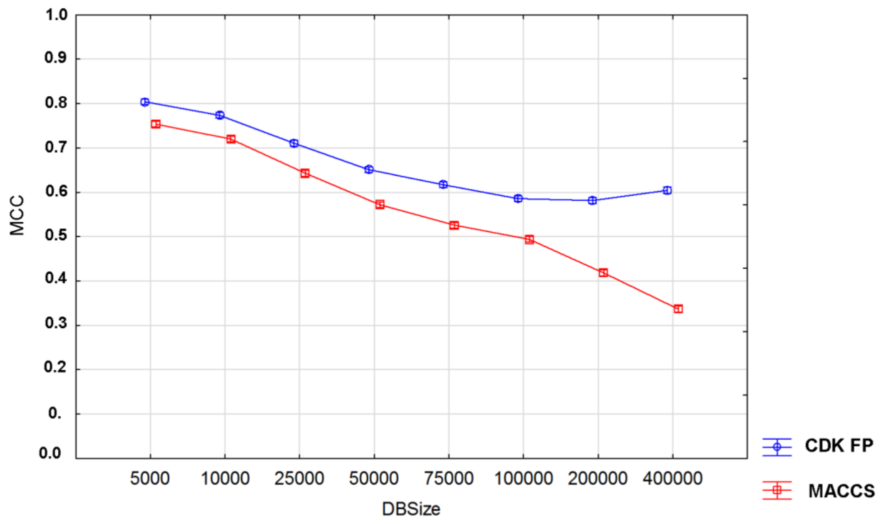

Analysis of Variance:

Source	SS	df	MS	F	p
FP	161.10	1	161.10	4017.8	0.0000
DBSize	614.30	7	87.76	2188.7	0.0000
FP*DBSize	62.15	7	8.88	221.4	0.0000
Error	2214.69	55234	0.04		

Case 5. Testing the effect of the screening library size on the virtual screening performance given by MCC value for different protein targets used.

Interaction plot:

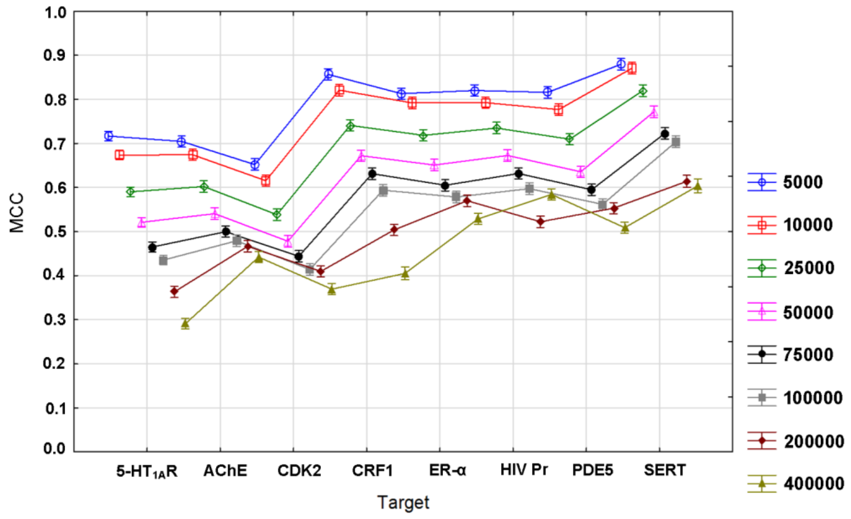

Analysis of Variance:

Source	SS	df	MS	F	p
Target	386.20	7	55.17	1512.8	0.0000
DBSize	625.66	7	89.38	2450.9	0.0000
Target*DBSize	33.98	49	0.69	19.0	0.0000
Error	2012.55	55186	0.04		
